# Seasonality of Leaf and Fig Production in *Ficus squamosa*, a Fig Tree with Seeds Dispersed by Water

**DOI:** 10.1371/journal.pone.0152380

**Published:** 2016-03-24

**Authors:** Pornwiwan Pothasin, Stephen G. Compton, Prasit Wangpakapattanawong

**Affiliations:** 1 Department of Biology, Faculty of Science, Chiang Mai University, Chiang Mai, Thailand; 2 School of Biology, University of Leeds, Leeds, LS2 9JT, United Kingdom; 3 Department of Zoology and Entomology, Rhodes University, Grahamstown, South Africa; 4 World Agroforestry Centre (ICRAF) c/o Knowledge Support Center for the Greater Mekong Sub-region (KSC-GMS), Faculty of Social Sciences, Chiang Mai University, P.O. Box 267, CMU Post Office, Chiang Mai, Thailand; Chinese Academy of Forestry, CHINA

## Abstract

The phenology of plants reflects selection generated by seasonal climatic factors and interactions with other plants and animals, within constraints imposed by their phylogenetic history. Fig trees (*Ficus*) need to produce figs year-round to support their short-lived fig wasp pollinators, but this requirement is partially de-coupled in dioecious species, where female trees only develop seeds, not pollinator offspring. This allows female trees to concentrate seed production at more favorable times of the year. *Ficus squamosa* is a riparian species whose dispersal is mainly by water, rather than animals. Seeds can float and travel in long distances. We recorded the leaf and reproductive phenology of 174 individuals for three years in Chiang Mai, Northern Thailand. New leaves were produced throughout the year. Fig production occurred year-round, but with large seasonal variations that correlated with temperature and rainfall. Female and male trees initiated maximal fig crops at different times, with production in female trees confined mainly to the rainy season and male figs concentrating fig production in the preceding months, but also often bearing figs continually. *Ficus squamosa* concentrates seed production by female plants at times when water levels are high, favouring dispersal by water, and asynchronous flowering within male trees allow fig wasps to cycle there, providing them with potential benefits by maintaining pollinators for times when female figs become available to pollinate.

## Introduction

The phenology of plants reflects selection generated by seasonal climatic factors, interactions with other plants and animals and constraints imposed by their phylogenetic history [1, 2]. Phenology is the study of the timing of life-history events associated with the course of the seasons and the factors that lead to its occurrence [3]. It influences evolution and adaptation at individual and community scales [4]. Studies of plant phenology have examined the timing of occurrence of biological events such as bud-burst, leaf-expansion, abscission, flowering, fertilization, seed set, fruiting, seed dispersal and germination [5, 6]. In tropical regions, climatic variations are less pronounced than elsewhere, leading to different plant responses in relation to climate. Seasons are often marked by differences in rainfall, with life-history events occurring in response to water availability [7]. The timing of biological often responds fluctuations of climate [8–10]. In general, the more specialized the relationship between species, the more vulnerable each of them is likely to be to the phenological effects of future climate change [4]. There is currently widespread concern that climate-driven changes in the timing of seasonal events may disrupt important ecological interactions such as pollination or cause temporal mismatches between critical periods in animal life cycles and food availability. More specifically, an extreme specialist such as fig trees of the genus of *Ficus* (Moraceae) have complex reproductive phenologies and exhibit one of the obligate species-specific mutualisms, with pollination performed by fig wasps (Agaonidae) [11–20]. Both the plants and their associated insects require the other for survival, to accomplish their life cycles. Together they form an obligate and specific mutualistic relationship and changes in the abundance of one of the partners can drastically reduce their fitness of the other [21]. The specific fig-fig wasp mutualism often follows locally a one-to-one rule (one Agaonidae species for one *Ficus* species) [22]. However, recent studies have shown that *Ficus* species often have more than one pollinator species in different geographical, and sometimes in sympathy [23–28]. Nevertheless, the fig and fig wasp association remains highly specific [29].

Seasonality in fig production has often been noted in phenological studies that report asynchronous flowering with continual fig production at the population-level, combined with flowering synchrony and sub-annual fruiting at the individual level [22, 30]. In general, the seasonal timing of flowering and fruiting, i.e. the reproductive phenology of plants, is determines the resources available to animals and consequently has effects on pollination and seed dispersal [31]. Flowering patterns may be related to the abundance of pollinators or the optimal time for pollination, and the ripening of fruits tends to peak during the best time for dispersal and germination [32]. Genus *Ficus*, with around 800 described species is divided reasonably evenly between monoecious and functionally dioecious species [33]. The breeding system of a fig species influences its reproductive phenology [34]. In monoecious *Ficus* species where each plant has figs that support seed production and the development of pollinator larvae each individual tree typically produces a few highly synchronous crops of figs each year, but asynchrony among trees allows year-round survival of the pollinating fig wasps populations [16].

In dioecious *Ficus* species, several crops of figs a year can be produced, but crops are often less synchronized than among monoecious species [17, 35, 36]. Relatively few studies have described the reproductive phenology of dioecious fig species in tropical region [34–38], but considerable variation in phenology is recorded [4, 35–37] with patterns often reflecting seasonal variation in climate [33]. Under subtropical to temperate conditions, male trees seem to produce a major annual crop of figs that releases pollinating wasps at the time when a major annual crop of figs on female trees is receptive, together with minor male crops that allow year-round survival of pollinating wasps [4]. For example, with *Ficus carica* L. in southern France, pollinators disperse between figs only twice yearly, in May and August [39]. Dispersal of the pollinator, *Blastophaga psenes* L. during the winter is thus avoided. In general, males of dioecious *Ficus* species produce figs all year round, more earlier [40]. Dioecy may be seen as a trait enabling male trees to support pollinators populations while allowing female figs to concentrate at times of optimal conditions, and at the same time avoiding self-fertilization [41].

In general, female fruit ripening peaks are expected to occur prior to favorable conditions for the germination of seeds and the development of seedlings [31]. Fruit development and seed maturation should be timed to match with the seasonal availability of legitimate dispersal agents and suitable environmental conditions for dispersal and plant establishment [42]. For these reasons, phenological synchronization among individuals of a population is directly related to the reproductive success of the species [43].

Thies and Kalko [44] have suggested that differences in the flowering phenology of tropical forests are primarily caused by abiotic climatic factors such as water and light, whereas differences in fruiting phenology are mostly influenced by biotic factors (the presence of seed dispersers). The flowering and fruiting phenologies of several dioecious *Ficus* species are related with seasonal conditions, such as: *Ficus auriculata*, *F*. *fulva*, *F*. *hispida*, *F*. *oligodon*, *F*. *semicordata*, *F*. *triloba* and *F*. *variegata*, in Thailand [45], *F*. *fulva* in Malaysia [38] and *F*. *variegata* in Australia [34].

Berg *et al*. [46] noted that *F*. *squamosa* has unusually long persistent styles with retrorse hairs which they regarded as adaptations to anchor the seeds to the substrate, allowing seeds to adhere to substrates on the soil or rock and preventing their washing-away into unfavorable sites. How this species disperse is unknown, but with regard to the seed morphological evidences and seed dispersal of rheophytes, water is expected to play a major role. Our others hypothesize that seeds of *F*. *squamosa* can float when it is ripening and falling into the water at times of high water level or floods. In several rheophytes, buoyant seeds have been observed, and some seeds have special adaptations to hydrochory (i.e. plant dispersal by surface water) [42]. Such adaptations can be: cork-like, air-filled tissue, resulting in a low relative density or a hydrophobic seed coat and fiber or hair covered seeds [42]. For example, seeds have outer layer with hooked fibers or hairs and may aid in dispersal by allowing the fruits to functions as trap air bubbles that float to the water flow and attached to seeds may be as anchors on the substrate (i.e. *Pinanga rivularis*, a rheophytic palm of Borneo [47] and *Euterpe endocarps*, a hydrophilic palm of Brazil [48]).

The natural hydrological conditions in rivers and streams are a prerequisite for facilitating the spatial movement of seeds especially during high floods. For example, Boedeltje *et al*. [49] suggested that water is a vector for transport of several type of seed plants and the majority of seeds was dispersed before late autumn and/or winter that floods may have transported seeds from streams into the canal at the Twentekanaal in the eastern part of the Netherlands. Besides Schneider and Sharitz [50] reported that the number of species and seeds dispersed were positively related to water levels in a swamp forest. The information about temporal dispersal pattern of *F*. *squamosa* in relation to discharge patterns carrying seeds is still poorly understood. It is, however, likely that surface water is an important vector for seed dispersal but it is not the only one dispersal vector in freshwater wetlands [42]. Although, there is no detailed information is available of the other dispersal vector of *F*. *squamos*a seeds, but wind and animal dispersal have an additional value to riverine plant seeds, besides hydrochory, whereas water dispersal in streams is unidirectional and takes place in downstream direction only, wind and animal may enable inter-catchment dispersal and upstream dispersal [42].

The seasonality of phenological events in riparian areas seems to be less pronounced. Whereas in some riparian forests the phenological events of the woody species are uniform along the year [7], in others they seem to be also associated with rainfall, with peaks in the wet season [51]. The present study aims to describe the reproductive phenology of riparian fig species and correlate them with climatic variables (i.e. air temperature, relative humidity, sunshine hours and rainfall).

We examined whether the riparian habitat of *F*. *squamosa*, and in particular whether its use of moving water to disperse its seeds, is reflected in its phenology of leaf and flower/fruit production. We addressed the following specific questions: (1) Do leaf and fig production vary seasonally in relation to climatic variables such as rainfall? (2) Does the fruiting phenology of *F*. *squamosa* reflect seasonal variability in water levels in its riparian habitat? and (3) Does the fruiting phenology of male and female plants differ? The results can help inform future management of water flows in SE Asian rivers, with implications for the conservation of riparian *Ficus* diversity [42].

## Materials and Methods

### *Ficus squamosa* and its fig wasps

Fig trees are abundant in wet lands and riparian forests of Thailand [52], but their phenological responses to seasonal variability are poorly understood. *Ficus squamosa* is a functionally dioecious species distributed along watercourses in tropical forests. It is strictly riparian species and rheophytic, restricted to streamsides, and is adapted to living and establishment in (rapidly) flowing water [46, 52]. It grows in riparian areas that are occasionally flooded, but may be dry for periods of the year [53]. In Northern Thailand, the water levels of streams in the rainy season can reach about 1–1.5 meters above these in the dry season (P. Pothasin, personal obs.). Plants such as *F*. *squamosa* are exposed to the highest water levels where become mostly submerged, and can even be destroyed by extreme floods. Normally, *F*. *squamosa* will hardly ever be submerged for such a long period of time (short or long heavy tropical rainstorms in the upper reaches of a stream may cause the water level to rise for several hours to a few days or during a longer rainy spell for a few weeks, but not usually for several months. When the flash floods pass and water levels go down again, the plants are re-exposed. Conversely, in the dry season, they are often growing in seemingly dry habitats such as cracks of rock, sandbars or sandy shores (P. Pothasin, personal obs.).

**Fig 1:**
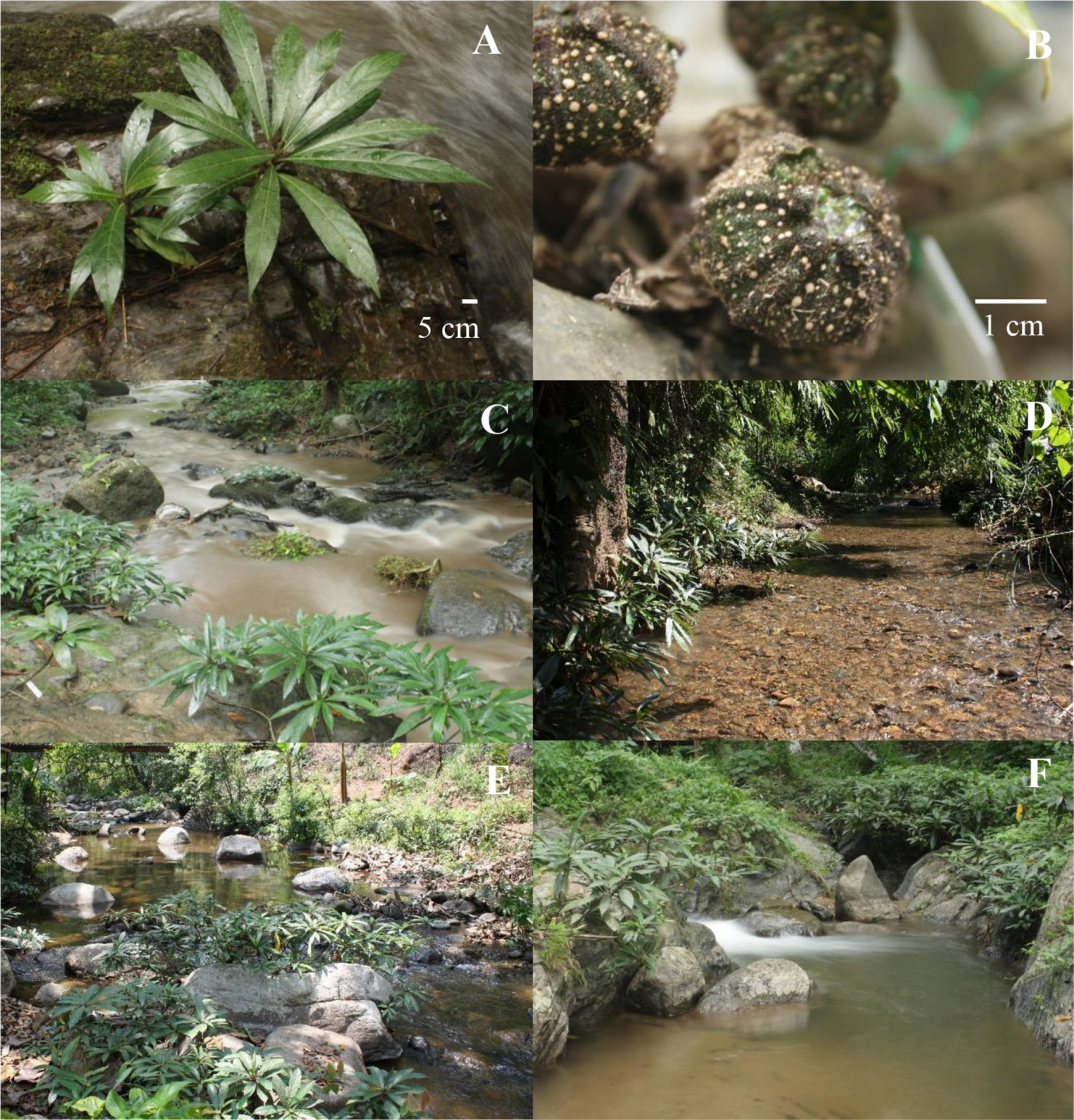
Species study and sample sites. (A) *Ficus squamosa* growth form, (B) figs, (C) Mae Ka site, (D) Pang Dang Nai site, (E) Mae Sa site, and (F) Mae Sa Noi site.

*Ficus squamosa* Roxb. (subgenus *Sycomorus*, section *Sycocarpus*) is a dioecious shrub with rooting stolon-like branchlets that grows approximately 1 to 2 m tall. It was placed in subsection *Macrostyla* by Berg *et al*. [54], together with a species from Sarawak, because they share flowers in their female figs that have very long persistent styles with deflexed hairs. The species are rheophytes and their unusual styles may be due to convergent adaptations to anchor water-borne fruits to river substrates [46]. The natural distribution of *F*. *squamosa* covers Thailand through to Laos, Myanmar, China (Yunnan), NE india, Sikkim, Bhutan and Nepal [55], where it is mostly found in the rocky beds of quick running streams, and no more than 10 meters the water’s edge [52]. Its figs are located along the branches and branchlets (Fig 1). The figs are rhombic-ovoid, reaching 3–4 cm in diameter at maturity. Both male and female figs remain yellow-green when ripe, reflecting their use of water currents (and possibly terrestrial mammals) for seed dispersal (P. Pothasin, unpublished data).

*F*. *squamosa* in northern Thailand is routinely pollinated by two *Ceratosolen* species and two undescribed non-pollinating fig wasps (NPFW), *Philotrypesis* sp. and *Apocrypta* sp., both presumed parasitoids of the pollinators, have also been reared from male figs of *F*. *squamosa* (S.G. Compton and P. Pothasin, unpublished data). As in most dioecious fig tree species, no fig wasp offspring were recorded from female figs.

### Study sites

Our study populations were located along perennial streams in the Ping River basin, Chiang Mai Province, Thailand (18° 51´ N, 98° 54´ E). The region is mountainous with some lowlands along river tributaries, with about 60 percent of the land area having an elevation above 500 m. Average annual rainfall, relative humidity, and annual temperature are 1073 mm, 70% percent, and 25.9°C, respectively [56]. The climate of Chiang Mai is influenced by tropical monsoons and falls into three main seasons, the rainy, the cool dry and the hot dry seasons (S1 and S2 Figs). Rainfall is frequent from June to late October. This is followed by the cool dry season until February, and the hot dry season running from March to June. Seasonality flooding is a regular feature of the monsoon climate. Heavy downpours are common in the rainy season or in September, and can cause flash floods. Floods are common in north Thailand because this region experiences the highest seasonality in rainfall distribution within Thailand and flash floods were reported for some area in Northern Thailand when monsoon rainfall completed with heavy rainfall [57].

Our four study sites (Fig 1) were in fragmented riverside forest [58–60]. The streams were fast flowing and bedrock-lined, with large boulders, (S1 Table). The streams flow all year and generally carry only small amounts of silt and sand. Runoff events and rapid temporary increases in flow rates occur in the rainy season, and result in water colour changing to reddish-brown. Two sites, Mae Ka (MK) and Pang Dang Nai (PDN) were located in the northern, Chiang Dao District of the Upper-Ping watershed and had mixed limestone and igneous substrates. The MK stream flows through Doi Chiang Dao’s wildlife Research Station, where some water emanates from a small natural hot spring. MK flows through hill evergreen forest, deciduous dipterocarp forest and agricultural land. The site, at an altitude of 480 m above sea level. The width of the stream was between about 3.5 and 12 m. The other two streams had mainly igneous substrates and widths ranging from approximately 5–20 m. The PDN stream flows through Ban Pang Dang Nai village and through maize fields and mixed deciduous forest with bamboos and Teak (*Tectona grandis* L.f.). This site, at an altitude of 500 m above sea level and a concrete check dam is present. Streams Mae Sa and Mae Sa Noi (MS and MSN) were located in the Mae Rim District and had several waterfalls. The sites, at an altitude of 350 and 570 m above sea level, respectively. Over the last decade much of their surrounding area has become peri-urbanized, with various tourist attractions and extensive stream-side encroachment.

### Meteorological data

Simultaneous meteorological data, including daily average temperature (in degrees Centigrade) and relative humidity (as a percentage), and sunshine (in hours of daylight) were obtained from the documentation of the Northern Meteorological Center, Thai Meteorological Department. Monthly rainfall (in millimeter) were obtained from the Hydrology and Water Management Centre for Upper Northern Region, Royal Irrigation Department, Thailand database. The weather data were measured at a fixed-site station located close to each site.

### Field observations

Leaves and figs on 174 mature *F*. *squamosa* individuals at the four sites were monitored every two weeks from April 2009 to early February 2011 (45 observations), with recording extended at MK and PDN to March 2012 to give a three-year observation period (giving 72 observations). Seventeen trees showed no evidence of fig production and were excluded from analyses. Several other trees were lost during flooding events.

The diameter and number of stems on each plant was recorded. For plants with more than one stem, a combined stem diameter was calculated from the sum of the basal areas of each stem. The height of each tree, its maximum crown width and the number of stems were also recorded. Leaf condition was summarized according to the following index: 0 (no leaves), 1(leaf buds), 2 (young leaves), 3 (mixed mature and young leaves), 4 (mixed mature and senescent leaves) and 5 (senescent leaves).

Crop durations were considered to start with the first fig to appear and end when the last fig disappeared. This temporal flexibility with respect to the timing if fruit crops appears to relate, at least in part, to the obligate mutualism of fig trees with wasp pollinators. Fig developmental phases were assigned based on the scheme of Galil [12]. A phase figs are immature, B phase figs are the stage when pollinators enter, C phase is when fig wasp offspring and seeds develop, D phase is when fig wasp offspring vacate male figs and E phase is when a mature female syconia with the internal space filled with seed. Prior to the main study, we collected samples of 10–20 figs at the four sites for dissection from individuals growing near to the focal trees to determine their stage of development in relation to fig diameter, while we used as an indicator for fig developmental phase. Since the size of figs is correlated with their developmental phases [11, 12].

On each occasion, we examined individuals for the presence of figs and recorded the numbers of figs present. Fig diameters were measured at their equator to the nearest 0.1 mm with Vernier calipers. We recorded the phase of development for the most abundant age class of figs on the tree, but also recorded any other phases present. Crop sizes were often small. We recorded crop sizes as the maximum number of figs present on a particular date and calculated only for crops, which finished development.

### Data analysis

Most statistical analyses were carried out in R (Version 3.2.0) [61]. As most data was not normally distributed and there was considerable variation in sample sizes, the non-parametric Mann-Whitney *U*-test was applied [62]. Similarity, for the analysis of distributions of the variance in the distributions of development phases among different groups, Kruskall-Wallis (K-W) non-parametric one way variance tests were used. Separate analyses were carried out for male and female trees growing at each site. We compared the size of plant (stem diameter, height and crown width) between male and female trees at each site. We also compared measures of fig abundance (fig production) and asynchrony using the pooled data. The presence or absence of asynchronous fig crops within individual trees was recorded with for example, an asynchronous crop in two or more development stage having figs within the same tree. Spearman rank correlations were performed between phenological and meteorological factors. Abiotic factors including sunshine hours, average mean temperature, average relative humidity and average rainfall were averaged over the 30 preceding days.

The reproductive phenological patterns of the plant and all circular statistical analyses were examined using ORIANA V.4 [63, 64]. We converted the dates of the phenological events to angles, from 0° (January) to 330° (December) at intervals of 30° per month [63, 65]. We estimated the mean angle (*a*), mean date (time converted from mean angle), vector length (*r*, concentration around mean angle) and circular standard deviation for each study site. Mean angle (*a*), and mean date, indicate average dates of the fruiting peak in the populations. Vector *r* has no units and indicates the degree of aggregation (seasonality) among individual plants fruit traps or synchrony of reproductive activity. High *r*-values generally indicate aggregated phenological behavior. We also performed a single-sample-distribution Rayleigh’s test (*z*) to determine whether the circular distribution of phenological activity was significantly non-random, or if there was seasonality. The Rayleigh test assessed the occurrence (or absence) of seasonal patterns within the four study sites. Mardia-Watson-Wheeler Multisampling Tests compared whether the variables had the same distributions throughout the year.

## Results

### Leaf phenology

**Fig 2:**
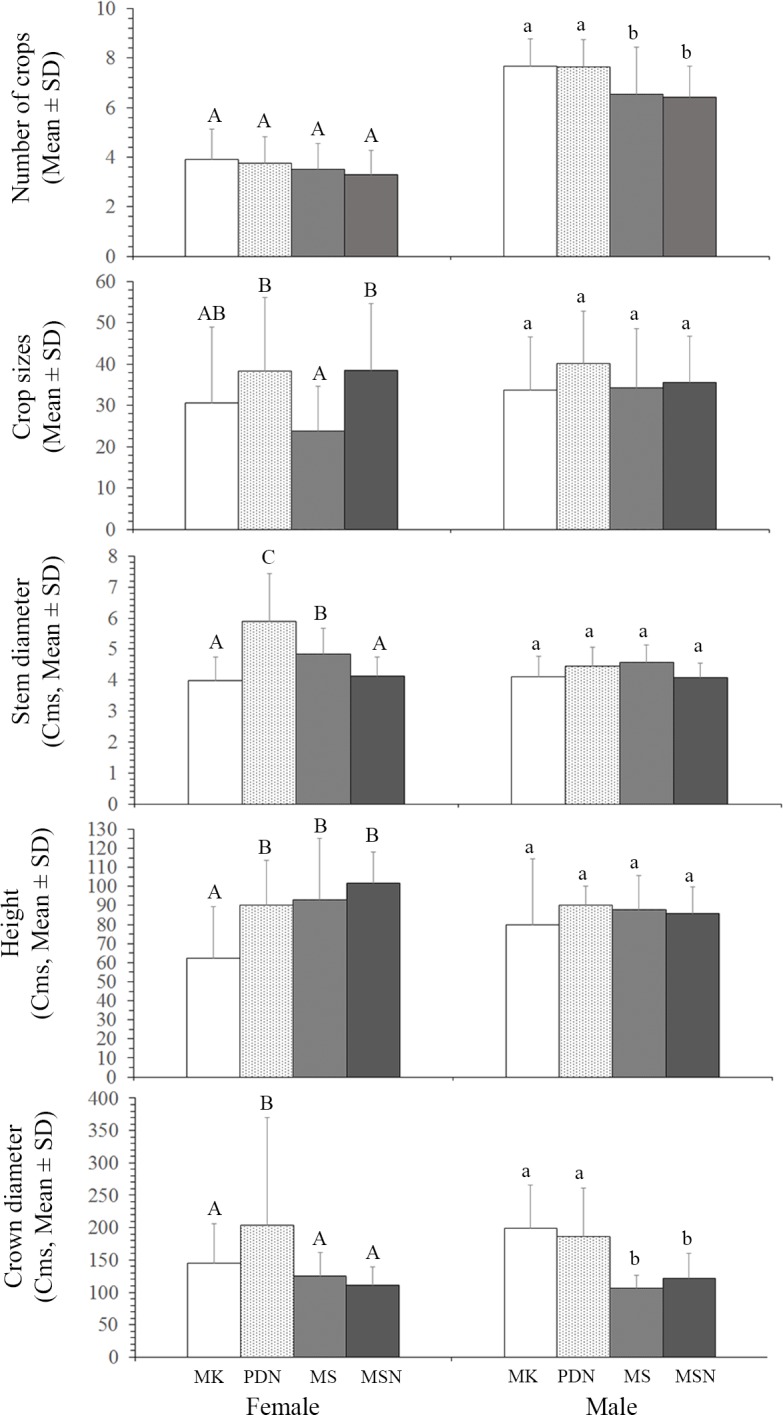
Bar graphs show the mean values and the standard deviations for each of the variables that showed statistically significant differences between study sites (MK, PDN, MS, and MSN). Different upper case letters on the bars represent significant differences among the means when Kruskal-Wallis and Mann-Whitney *U*-test.

Of the tree characteristics measured, the mean of stem diameter (d.b.h.), height of trees and crown diameter were significant differences between sites in female trees, but not in male trees except crown diameter (Kruskal Wallis test, female trees: stem diameter: *x*^2^ = 41.58, *P* = 0.000, n = 120; height of trees: *x*^2^ = 46.09, *P* = 0.000, n = 120; crown diameter: *x*^2^ = 7.57, *P* = 0.056, n = 120, male trees: stem diameter: *x*^2^ = 6.42, *P* = 0.093, n = 54; height of trees: *x*^2^ = 3.84, *P* = 0.280, n = 54; crown diameter: *x*^2^ = 20.64, *P* = 0.000, n = 54, Fig 2).

**Fig 3:**
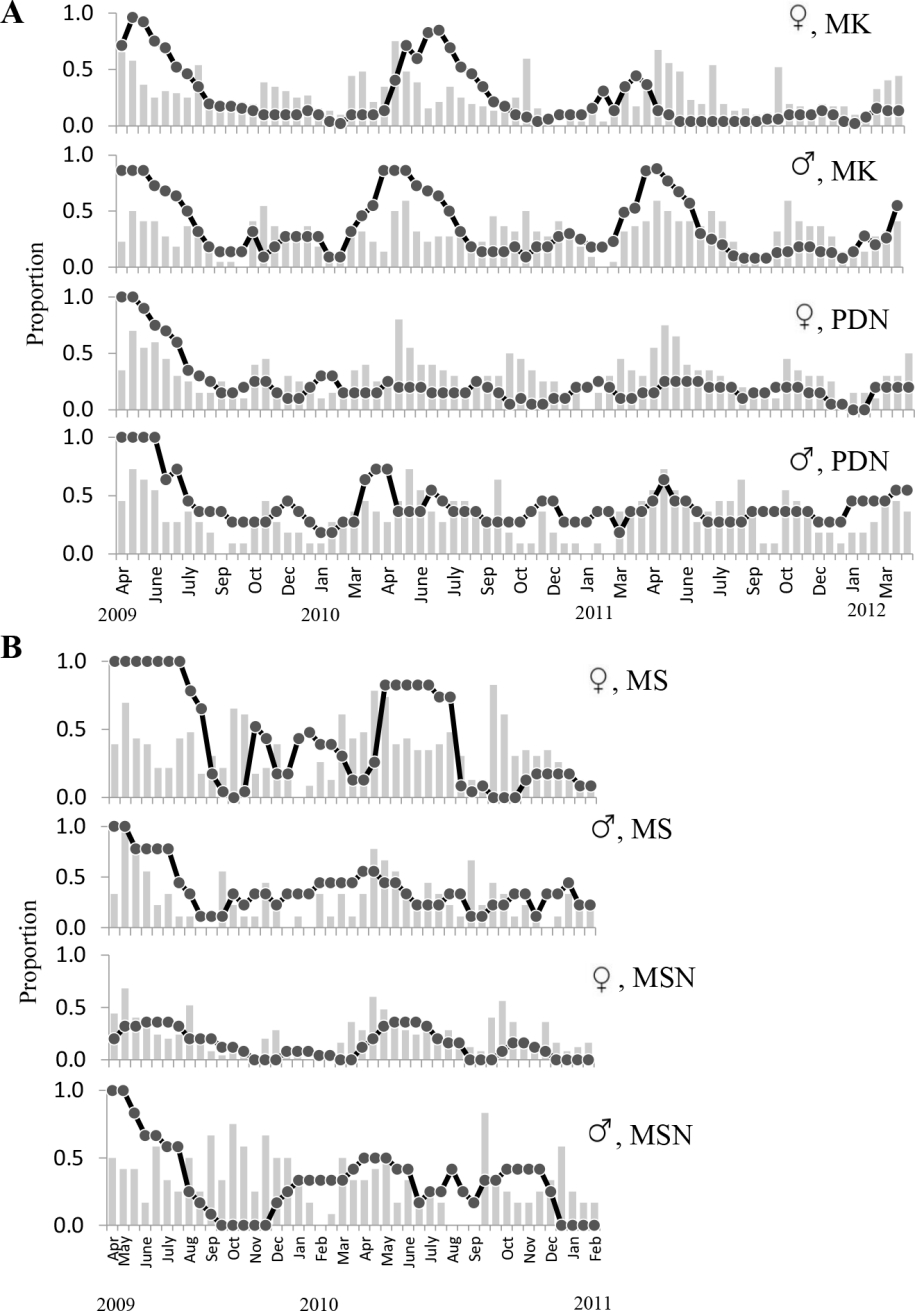
Seasonal changes in the proportion of *F*. *squamosa* with a mixture of young and mature leaves (grey bars) and with figs present (circles). (A) sites MK and PDN; (B) sites MS and MSN.

Both male and female *F*. *squamosa* were evergreen at our study sites, with small-scale leaf loss and gains of new leaves occurring almost throughout the year. Newly opened leaves took about one month to mature. Based on synchronized flashed of leaves, plants that produced well. The average longevity of the leaves varied between 6–8 months on different plants. The fewest leaves were present on the plants in the dry season (January to April) and late September, with peak numbers of new leaves in May, after rains had ended the dry season (Fig 3).

**Fig 4:**
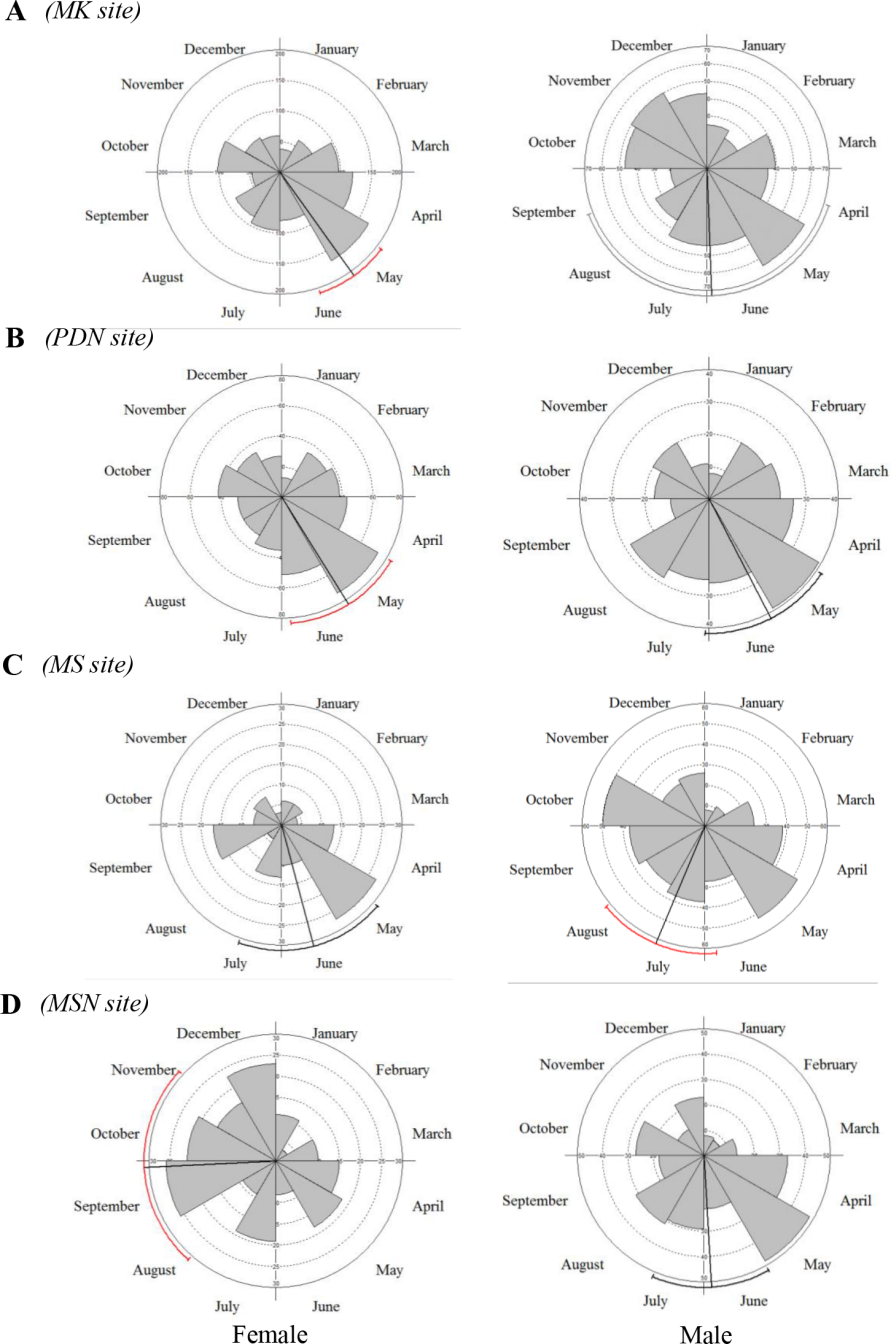
Circular histograms at monthly intervals of the numbers of *F*. *squamosa* bearing a mixture of young and mature leaves. Note that axis scales vary between histograms. The vector line represents the mean axis and the arc outside the circle is the 95% confidence interval for the mean axis (light arcs indicate the estimated of the confidence interval is unreliable).

New leaf production on a few plant was a little bimodal, about 6 months apart, but most plants revealed a single seasonal peak in new leaf initiation (Fig 4). Rayleigh’s Z values for seasonality of leaf initiation were mostly highly significant (Rayleigh tests, *P < 0*.*001*, S2 Table). Z values were consistently lower among males than female plants, and at two sites compared with the other sites. There was no significant seasonality in the occurrence of young leaves among male plants. In female trees, leaf initiation was concentrated in late May at MK and PDN, in the middle of June at MS and at the end of September at MS. In male trees, leaf initiation peaks occurred from May to July at the different sites (Fig 4).

New leaf production of both sexes was significant positively correlated with higher temperatures and negatively correlated with relative humidity (Table 1). The proportion of trees with young leaves present was mostly significantly correlated with the proportion of fig-bearing trees and new leaves were sometimes significant positively correlated with numbers of figs initiated (Table 1).

**Table 1:** Correlations between proportion of plants with new leaves, fig production and meteorological factors for *Ficus squamosa*.

	MK		PDN		MS		MSN	
	Female	Male	Female	Male	Female	Male	Female	Male
	(n = 52)	(n = 22)	(n = 20)	(n = 11)	(n = 25)	(n = 12)	(n = 23)	(n = 9)
Average Temp.	0.437**	0.344**	0.439**	0.482**	0.598**	0.479**	0.571**	NS
Minimum Temp.	NS	NS	0.261*	0.309**	0.487**	0.433**	0.514**	NS
%Rh	-0.353**	-0.303**	-0.370**	-0.420**	-0.322*	-0.315*	-0.302*	NS
Sunshine	NS	NS	NS	NS	NS	NS	NS	NS
Rainfall	NS	NS	NS	NS	NS	NS	NS	NS
Fig-bearing trees vs New leaf	0.076*	0.061*	-0.102*	0.110*	-0.134*	-0.014*	0.335*	-0.016*
Figs vs New leaf	0.289*	NS	NS	0.241*	NS	NS	0.587**	NS

Spearman’s rho (rank correlation tests) between the proportion of *F*. *squamosa* bearing a mixture of young and mature leaves and different abiotic factors at monthly averaged were calculated. “NS” indicates they were non-significance.

NS P > 0.05

*P < 0.05

**P < 0.01 (Spearman's rank correlation test).

### Reproductive phenology

The plants were assessed at approximately two-weekly intervals from April 2009 to March 2012. Eighteen plants that produced no figs were excluded from further analysis. Eleven of the initial 174 plants were lost through flash floods and associated erosion of the stream banks during the course of observation. Mature figs were retained on female plants until they were carried away by rising water levels during the rainy season and did not become noticeably softer or changes colour before being carried away.

Fruiting patterns were broadly similar at the four sites. A total of 442 crops were recorded from 120 female plants and 389 crops from 54 males. Individual female trees produced up to 6 completed crops and averaged 3.68 ± 1.12 crops (mean ± SD) crops during the period of the 36-month (n = 120 trees), equivalent to 1.43 crops per year. Male figs produced 3–9 completed crops with an average of 7.20 ± 1.38 (mean ± SD) crops (n = 54 trees), equivalent to 2.82 crops per year. There were no significant differences between sites in the number of crops produced by each sex over the study period (Kruskal Wallis test, female trees: *x*^2^ = 6.330, P = 0.097, n = 120; male trees: *x*^2^ = 8.867, P = 0.031, n = 54).

The average crop sizes were much higher on male trees at the four sites (female trees: mean ±SD = 32.27 ±18.45, n = 442 crops; male trees: mean ±SD = 37.83 ±18.45, n = 389 crops). However, there were no significant differences in the average crop sizes between sexs (Mann-Whitney *U*-test, U = 0.918, *P* = 0.359). Further analysis of site by site comparisons of reproductive phenology showed that the average crop sizes were significant differences between sites in female trees, but not in male trees (Kruskal Wallis test, female trees: *x*^2^ = 12.87, *P* = 0.005, n = 120; male trees: *x*^2^ = 2.03, *P* = 0.566, n = 54.)

Tree diameter (d.b.h.), height of tree and crown diameter were all not significantly correlated with the number of crops produced over the study period by each sex (Spearman rank correlation tests; Table 2). Crop size of male and female trees were both significantly correlated with d.b.h., but there were not significantly correlated with height and crown diameter except in MK site, where crop size was significantly correlate with height of tree (Spearman rank correlation tests; Table 2).

**Table 2:** Spearman rank non-parametric bivariate correlation between number of crops and crop size with stem diameter, height and crown diameter in each site and sex.

		no.of crop		crop size		no.of crop		crop size	
		female				male			
		r	*p*	r	*p*	r	*p*	r	*p*
**MK**	d.b.h.	-0.111	0.432	0.994**	0.000	-0.142	0.527	0.992**	0.000
	height	0.178	0.207	0.534**	0.000	-0.201	0.370	0.662**	0.001
	width	0.061	0.666	0.132	0.350	0.194	0.387	-0.280	0.901
**PDN**	d.b.h.	-0.258	0.271	0.984**	0.000	-0.051	0.882	0.953**	0.000
	height	-0.210	0.374	-0.005	0.985	0.289	0.388	-0.430	0.187
	width	-0.840	0.726	0.393	0.087	0.253	0.452	0.239	0.480
**MS**	d.b.h.	-0.231	0.195	0.892**	0.000	0.190	0.624	0.940**	0.000
	height	0.246	0.156	-0.650	0.684	-0.195	0.615	-0.430	0.248
	width	-0.270	0.111	0.327	0.035	-0.254	0.509	-0.299	0.434
**MSN**	d.b.h.	-0.630	0.765	0.937**	0.000	0.302	0.340	0.893**	0.000
	height	-0.298	0.149	0.220	0.290	-0.548	0.065	-0.302	0.341
	width	0.100	0.635	0.161	0.443	-0.599*	0.040	-0.258	0.419

**. Correlation is significant of the 0.01 level (2-tailed).

*. Correlation is significant of the 0.05 level (2-tailed).

**Fig 5:**
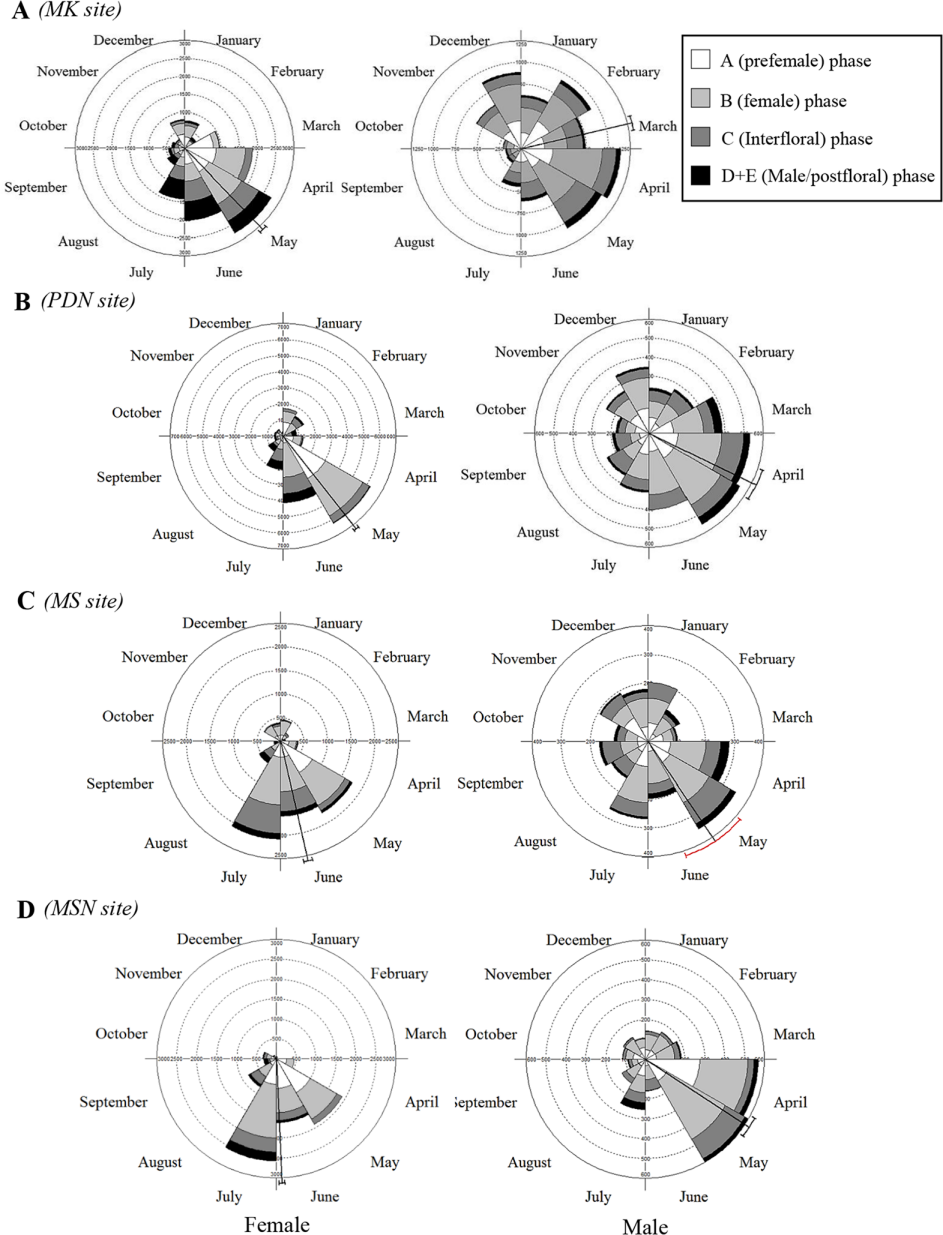
Circular histograms, vectors and distributions of figs at different developmental phase (A-D) at four study sites. The vector represents the mean bearing and the arc outside the circle is the 99% confidence interval for the mean bearing.

*F*. *squamosa* individuals initiated fig crops at different times throughout the year, with obvious seasons when few or many figs were present. Sexual differentiation in phenological patterns was apparent. More crops of male trees were asyschronous than female trees (define what asynchronous was = B and D+E stages) (Mann-Whitney U-test, P < 0.001; Table 3). *F*. *squamosa* producing figs all year-round, more or less continuously on male trees than female trees (As an example at MK site, female; S3 Fig, Male; S4 Fig). Some crops on both male and female trees were aborted without having been entered by pollinators. Crop abortion was more common on female than male trees (e.g. female tree; numbers 9, 10, 13–15 and 35). A few female trees (numbers 1, 2, 3, 13, 16 and 37) died because of the flooding after the rainy season in 2010. At the population level, the flowering onset of female trees did not occur randomly over the year, as new crops initiations were seasonal (Rayleigh tests, *P* < 0.001, S3 Table). The timing and degrees of seasonality in the peaks of fig initiation (mean vector lengths) differed between sites. Most female trees at HMK and PDN initiated figs on May, about a month earlier than MS and MSN. Fruiting in male trees was generally distributed more uniformly, with only slight seasonal highs and lows (S3 Table, Fig 5). Fig developmental phases of *F*. *squamosa* were assigned based on fig diameter, since the size of figs is roughly correlated with the development phases by random dissection 20 figs per sex/site. A phase figs are immature or early floral development with size 0.3–1.7 mm in female trees and 0.3–2.0 in male tree. B phase figs are the stage when pollinators enter (oviposit) with size 1.7–2.5 in both sexes. C phase is when fig wasp offspring and seeds develop with size1.7–3.0 in female trees and 1.7–3.5 in male trees. D phase is when fig wasp offspring vacate male trees and the figs turned to yellow or light green colour with size 1.9–3.5 mm. E phase is when a mature female fig with the internal space filled with seed and turn to yellow or brown with size 2.0–4.0 mm.

**Table 3:** Mean number of figs, crop sizes, asynchrony, stem diameter, height and crown diameter per tree in each site over the study period.

		female			male			*P*
		n	Mean	S.D.	n	Mean	S.D.	
**MK**	The number of crops	52	3.92	1.20	22	7.68	1.09	0.000*
** **	Crop size	204	30.63	18.31	169	33.77	12.83	NS
	Asynchrony	385	0.10	0.11	566	16.97	14.85	NS
** **	Tree diameter (d.b.h.)	52	3.98	0.77	22	4.11	0.66	NS
** **	Height of tree	52	62.35	27.12	22	79.95	34.53	NS
** **	Crown diameter	52	145.08	60.88	22	198.91	67.36	0.003*
**PDN**	The number of crops	20	3.75	1.07	11	7.63	1.12	0.000*
** **	Crop size	75	38.23	17.94	84	40.12	12.72	NS
	Asynchrony	209	0.15	0.16	224	0.28	0.15	0.000*
** **	Tree diameter (d.b.h.)	20	5.88	1.55	11	4.45	0.61	0.001*
** **	Height of tree	20	90.00	23.84	11	90.00	10.00	NS
** **	Crown diameter	20	204.00	166.30	11	186.36	74.74	NS
**MS**	The number of crops	23	3.52	1.04	9	6.55	1.88	0.000*
** **	Crop size	81	23.88	10.72	59	34.21	14.32	NS
	Asynchrony	171	0.17	0.18	110	0.27	0.17	0.003*
** **	Tree diameter (d.b.h.)	23	4.83	0.83	9	4.56	0.58	NS
** **	Height of tree	23	93.04	31.97	9	87.78	17.87	NS
** **	Crown diameter	23	125.22	36.91	9	106.67	19.36	NS
**MSN**	The number of crops	25	3.28	0.98	12	6.42	1.24	0.000*
** **	Crop size	82	38.43	16.15	77	35.53	11.24	NS
	Asynchrony	67	0.06	0.07	125	0.23	0.21	0.000*
** **	Tree diameter (d.b.h.)	25	4.14	0.61	12	4.07	0.48	NS
** **	Height of tree	25	101.60	16.25	12	85.83	13.79	NS
** **	Crown diameter	25	111.40	28.27	12	121.67	38.96	NS

n = sample size of female and male trees. Asterisks imply significances between means from pairwise comparisons of sexes using the Mann-Whiney *U*-test.

### Correlation with meteorological factors

The numbers of A-phase (recently initiated) figs and the total numbers of figs on the plants were correlated with meteorological data. Generally, A-phase figs and total figs were positively correlated with mean temperatures over the previous 30 days, but figs on male trees at MK had a significant negative correlation with temperatures (Table 4). Mean minimum temperatures were negatively correlated with total male fig numbers at MK and PDN, but positively correlated with fig numbers on female trees at MK, MK and MSN (Table 4). Sunshine hours were positively correlated with total fig numbers of both sexes at MK but was only positively correlated with total fig numbers on male trees at PDN, MS and MSN. Relative humidity was negatively correlated with total fig numbers on both plants of both sexes at every site. Finally, rainfall was negatively correlated with the numbers of figs on male trees at all four sites, but positively correlated with fig numbers on female trees at PDN and MSN (Table 4).

**Table 4:** Correlations between phenological and abiotic factors in different *Ficus squamosa* populations.

	MK				PDN				MS				MSN			
	Female		Male		Female		Male		Female		Male		Female		Male	
	A	Tot.	A	Tot.	A	Tot.	A	Tot.	A	Tot.	A	Tot.	A	Tot.	A	Tot.
**Average air temp (°c)**	+	+	-	-	NS	+	NS	+	NS	+	NS	+	+	+	NS	+
**Minimum air temp (°c)**	+	+	-	-	NS	NS	NS	-	+	+	NS -	NS	+	+	NS	NS
**Relative humidity (%)**	-	-	-	-	-	-	-	-	-	-	-	-	-	-	-	-
**Sunshine hours**	+	+	+	+	NS	NS	+	+	NS	NS	+	+	NS	NS	+	+
**Rainfall (mm)**	NS	NS	-	-	NS	+	NS	-	NS	NS	NS	-	+	+	NS	-
**Rainfall 30 day tot. (mm)**	NS	NS	-	-	NS	NS	NS	-	NS	NS	NS	NS	NS	NS	NS	NS

Spearman’s rho (rank correlation tests) between numbers of A-phase figs (A) or total numbers of figs (Tot.) and different abiotic factors averaged over the 10, 20, 30, 60 preceding days, and with a 10-day delay were calculated. “+” indicates that some correlations over different durations were positive and significance (*P* <0.05). “-” indicates they were some negative and significance (*P* <0.05) and “NS” indicates they were all non-significance. We never obtained a mix of significant positive and negative correlations. The full results are available in the supplementary data (S4 Table).

## Discussion

Studies conducted in riparian forests point out a phenological pattern of *F*. *squamosa* which is one of the dominant fig species in stream habitat in the northern part of Thailand [52]. *Ficus squamosa* is a shrub with creeping stem found in rocky beds of fast-flowing streams that are exposed to two alternate environments. During summer period the water level is low, the species are exposed to dry air and high temperatures. On the other hand, during rainy season, water level rises and flash floods occur frequently, so these plants are exposed to severe flooding periods, with bank erosion and burial sediment making it inaccessible. The species that occupied these habitats developed unique characteristics as well, due to the fact that they must stand the flood period. Nomura *et al*. [66] noted in general that the narrow shape of the leaves or rheophytes is the adaptation not for the light intensity but for the flood resistance, through an increased mechanical toughness. *F*. *squamosa* has figs that originate on branches near to the water level or even below. Their fruits have brushy long, hairy stigmas, which persist when the figs are ripe. Seeds can also take advantage of water currents to float their offspring away. This is an adaptation for growing in running streams near fast-flowing rivers [54].

*Ficus* was unique in that the association between *Ficus* (Moraceae) and their pollinators, the fig wasps (Agaonidae, Hymenoptera), involves a species-specific and unique pollination system [11, 67]. Phenology of flowering production at the population level must ensure survival of the pollinators if their obligate mutualistic relationship is to be maintained. This requirement may bring about the typical phenological pattern of *Ficus* found in tropical regions, which is annual or supra-annual flowering at the individual level integrated into a continuous pattern at the population level [33]. Fig production in *F*. *squamosa* was concentrated in the hot season of the year. A single annual generation of pollinators emerged from *F*.*squamosa* male figs at a time when some conspecific female figs were receptive, so these figs could be entered by the fig wasps carrying *F*. *squamosa* pollen and seed set could develop. Their phenologies are nonetheless responsive to climatic variations and the tropical monsoon climate in northern Thailand appears to generate phenological patterns in *F*. *squamosa* growing in the stream habitat that support the maintenance of populations of it associated fig wasps.

### Leaf phenology

The production of leaves on *F*. *squamosa* trees in northern Thailand varied among individuals, but there was a clear response to the individuals of *F*. *squamosa* showed evergreen behavior. *F*. *squamosa* never appears leafless during the entire year. Although some new leaves were produced in all months of the year, they were more abundant in the beginning wet period (May), greening the canopy well in advance of the onset of the monsoon. Leaf exchange occurred in small amounts throughout the year, leaf senescence and leaf fall was maximal in the dry season (January to April) and no differences between the sexes. *F*. *squamosa* in southwestern China by Liu *et al*. [68] exhibited similar leaf phenology in our study. There was little seasonal variation in the leaf states, with small quantities of new and senescing leaves present more or less continuously (see citations in figure 2 of Liu *et al*. [68]). Similar observation has also been reported in other species by kuaraksa *et al*., reported that *F*. *semicordata* and *F*. *hispida* were evergreen in dry deciduous forest to hill evergreen forest in northern of Thailand [45].

The pattern of leaf phenology was strongly influenced by drought. Drought caused a significant increase in leaf shedding which was then followed by new leaf production with the renewal of rain. Harrison *et al*. reported a sudden increase in the deciduousness of fig species that was associated with severe drought linked to the El Niño event of 1997–1998, thus suggesting that the pattern of leaf phenology in figs was strongly influenced by drought and major rainfall oscillations may substantially affect phenology [38]. In seasonal environments, deciduous species may or may not initiate growth before evergreen species, but they complete it before the evergreens. They shed all their leaves prior to dry winter, which may allow them to conserve and store water and survive during cold dry winters [69, 70] (i.e. *F*. *variegata* [34, 45], *F*. *obtusifolia* [71], *F*. *racemosa* [72], *F*. *fulva* [38, 45], *F*. *auriculata*, *F*. *oligodon* and *F*. *triloba* [45]). In addition to the degree of drought to which trees are exposed varies widely, depending on temperature and availability of soil water, and tree characteristics such as rooting depth [31]. Accordance with the reports from other monsoon forests in Thailand, deciduous species form new leaves one to two months before the first monsoon rains in April/May, during the hottest and driest part of the year around the spring equinox. While evergreen species are restricted to relatives moist and there were almost no discernable seasonality of vegetative development [73]. Moreover, most of evergreen species shows deep root system, enabling access to water [74]. As rheophytes, *F*. *squamosa* shows stolon-root like stem system, so they were maintained a high water potential during the dry season.

We found that the proportion of trees with young leaf presents was mostly significantly positively correlated with temperature in both sexes, but neither between young leaf present and rainfall in both sexes. In tropical trees initiation of leaf flush (leaf bud), marking the termination of deciduousness duration, has been reported to be triggered by several factors such as increasing day length and/or temperature, significant amounts of the first rain, and photoperiod [74, 75]. According to Borchert [76], the increased photoperiod with rising temperature may cause conversion of starch into sugar in the roots and stem and osmotic adjustment in bud tissues which may induced bud busting by increasing water absorption and availability of sugars in summer flushing trees. However, there was a slight negative correlation with leaf flushing and relative humidity. In *F*. *squamosa*, drought caused a significant decrease in leaf flushing which proportion of trees without young leaves visibly matched with dry periods.

Correlations between vegetative and reproductive phenologies occurred in some figs, such as *F*. *fulva* in lowland dipterocarp forest at Sarawak, leaf flushing correlate with syconia initiation almost certainly reflects a physiologically based constraint on species, which produce their syconia at the tips of the twigs [38] and *F*. *variegata* in a seasonal wet tropical forest at caper tribulation, Australia [34]. In *F*. *squamosa* showed that before initiation of the peak fig crops, most female trees initiated new leaves, and then produced new figs simultaneously, or fig initiation occurs soon after leaf flushing, to ensure that they have enough fresh energy sources to support development main seed crop. Leaf flushing or bud burst were correlated with syconia initiation in female trees at MK and MSN sites and male trees at PDN site. However, this temporal sequence is not universal among figs.

### Fig phenology

*F*. *squamosa* population along riparian areas in Chiang Mai produces fig continually and the reproductive phenologies of male and female trees were different. Most of the study trees did not produce figs (10% of all trees observed have never produced figs), although all individuals were apparently mature (mean stem diameter >3 cm). The population-level phenology of *F*.*squamosa* is similar to the phenological pattern for dioecious figs proposed by kjellberg and Maurice [41], patterns that were also seen at the population level [36] and parallels the patterns of *F*. *carica* (subg. *Ficus*, sect. *Ficus*) in southern France [4], *F*. *variegata* (subg. *Sycomorus*, sect. *Sycomorus*) in a seasonal wet tropical forest at Cape tribulation, Australia [34], *F*. *hispida* (subg. *Sycomorus*, sect. *Sycocarpus*) and *F*.*semicordata* (subg. *Sycomorus*, sect. *Hemicardia*) in Doi Suthep-Pui National Park, Northern Thailand [45]. Kjellberg and Maurice proposed that dioecious figs respond differently to seasonal variability than monoecious figs, due to the separation of seed and wasp production between trees [41]. In dioecious *Ficus*, each gender specializes in making ether seeds or wasps, but wasp production translates directly into male reproductive function since wasps are the pollen vectors. The female figs would be produced during the wet season, presumably under the most favorable conditions for seed production, germination and establishment. The crop development period was approximately twice as long on female trees. On the other hand, male trees maintain a local wasp population by producing figs during adverse conditions. This pattern benefits male individuals (not simply the population as a whole) by increasing their reproductive success through the donation of pollen to females [16]. At the population-level, *F*. *squamosa* displayed asynchronous fig production. Even figs were produced year-round, but there were pronounced annual cycles in fig abundance. As fig mutualistic pollinators have a short lifetime (up to 2 days) [4], inter-individual flowering asynchrony is essential for the reproduction of the pollinating fig wasps and consequently, for the success of the fig-fig wasp mutualism [19]. However, our results pointed out seasonality in fig abundance that was significantly correlated with climatic seasonality (S3 Table). It is important to stress that riparian areas in northern of Thailand have a typical moonsoon climate and presents temperature and rainfall seasonality [73]. There are occasionally flooded during rainy season and exposed to dry air and very high temperatures when the water is low in hot season [77]. Male trees have shown sub-annual flowering patterns and produced larger productions of figs during hot season (April–May) in all sites. Female trees have an annual flowering pattern during late hot season to rainy season (May–July). We suggest that continuous production of figs is an adaptation to the stream habitat conditions. In addition, we observed fig abundance on female trees was significantly positively correlated with rainfall, but for male trees fig abundance showed a significant negative relationship with rainfall.

*F*. *squamosa* demonstrated considerable sexual specialization. At the individual-level, male *F*. *squamosa* trees produce a greater proportion of asynchronous crops than females. We observed the initiation of fig production showed seasonality in female trees, but mostly male trees appeared to be random or aseasonal in the initiation of fig production (S3 Table). Non-seasonality could be an adaptive feature, as Kjellberg and Maurice [41] have demonstrated with simulations that the number of plants needed to support the pollinator increase with seasonality reproduction.

We observed a peak in male fig production preceding the wet season (April—May) can increase the pollinator population, releasing pollen-carrying wasps at a time when the majority of female figs are receptive to pollinators. We may compare these results with results obtained on *F*.*hispida* [45], *F*. *fistulosa* [78], and *F*. *septica* [79], three species that belong to the same species group as *F*. *squamosa* within section *Sycocarpus* [54]. While fig production is rather continuous throughout the year, the curve of increasing male fig production preceded the curve of increasing female fig production, suggesting adjustment to pollinate female trees.

In addition to the tendency for males to produce more crops per year than females, which produces three to nine crops of wasps and one to six crops of seed figs per year. *F*. *squamosa* have also tended to demonstrate a longer development time of syconia on female trees than on male trees. Patel and Mckey [36] suggested that the longer development times for female figs were because of the greater number of ovaries and fleshier figs on female trees requiring greater investment of resources. This pattern differs from that of dioecious *F*. *auriculata* (subg. *Sycomorus*, sect. *Sycomorus*), *F*. *fulva* (subg. *Ficus*, sect. *Eriosycea*) and *F*. *triloba* (subg. *Ficus*, sect. *Eriosycea*) in Northern Thailand [45]. In fact, tropical phenological patterns can vary widely over the geographic range of species [32]. Even the phenological pattern of *F*. *squamosa* is similar to *F*. *variegata* in Australia [34], but differs from that *F*. *variegata* in Northern Thailand [45]. It would therefore be instructive to compare the phenological patterns of dioecious in seasonal wet tropical forest and the dry monsoon forest parts of its range. Kuasaksa [45] reported that *F*. *variegata* in mixed forest (evergreen and deciduous) in Thailand was synchronous within-trees of both sexes. However, there was displayed asynchronous fig production at the population level in both areas [34, 45].

## Conclusion

In Northern Thailand, *F*. *squamosa* is typically a species of riparian habitats, where is exposed to two alternate environments; i.e. the dry air and very high temperatures during dry period when the water level is low and the flooding period during rainy season. The present study of the phenology of *F*. *squamosa* demonstrated considerable sexual specialization and adaptive to riparian habitat. At the population level, male *F*. *squamosa* tree bears figs continually to maintain the pollinator fig-wasps population, while female tree produces one to several fig crops during the optimal season for seed production, dispersal and establishment (rainy season). The timing of dispersal of *F*. *squamosa* is shaped by the arrival of the rainy season and water vectors seem to be the main factor of seed dispersal their local habitats.

Fig abundance distributed non-randomly or seasonality over the year (Kuiper’s test, *P <* 0.001), with moderate concentration during the hot season in male trees and rainy season in female trees. Continual male fig production at population level is a consequence of flowering asynchrony at the individuals, but we don’t know exactly with which development phases overlapped within tree. Incidentally, this continuous fig production and within-tree asynchronous in male figs also allows survival of pollinators within riparian habitat, with unpredictable unfavorable growth conditions due to occasionally flash floods and may be dry for varying portion of the growing season.

However, *F*. *squamosa* had high ability to maintain their pollinator populations all year-round, even a few trees were lost through flash flood and bank erosion effect during rainy months. Figs were initiated rapidly after flash flood damage. We observed increasing of male fig production preceded the curve of increasing female fig production. The annual peak in male fig ripening (and wasp-release) was synchronous with the annual peaks in female fig receptivity, suggesting adjustment to pollinate female trees. This ensures enough pollinators for pollination success and avoids possible pollinator competition when wasps are unable to discriminate between the sexes of trees.

## Supporting Information

S1 Fig Monthly average minimum (grey lines) and mean (black lines) air temperatures and sunshine hours. (A) the northern site; Mae Ka (MK) and Pang Dang Nai (PDN); (B) the sourthern sites Mae Sa (MS) and Mae Sa Noi (MSN). (Source: Thai Meteorology Department, 2012, Available: http://meteorology.hrdi.or.th/website, accessed 26 February 2013).
(TIF)

S2 Fig **Monthly average rainfall and relative humidity; A, at Mae Ka (MK) and Pang Dang Nai (PDN); B, at Mae Sa (MS) and Mae Sa Noi (MSN)**; **Circular histograms distributions showing the mean and distribution of rainfall in the study sites; C, MK and PDN sites; D, MS and MSN sites.** (Source: Thai Meteorology Department, 2012, Available: http://meteorology.hrdi.or.th/website, accessed 26 February 2013).
(TIF)

S3 Fig Fig crops on 52 female *F*. *squamosa* at Huay May Ka (MK) from April 2009 to March 2012. (Hatched bars is aborted crop and ‘x’ is the tree lost during the period of study)
(TIF)

S4 Fig Fig crops of 22 male *F*. *squamosa* at Huay May Ka (MK) from April 2009 to March 2012. (TIF)

S1 Table Geological and environmental characteristics of the study sites. (DOCX)

S2 Table Results of circular statistics analyses testing for the occurrence of seasonality in leaf initiation of *F*. *squamosa* at four sites. Mean angle (*a*), and mean date, indicates the months of the leafing peak. Vector *r* indicates the extent of synchrony of reproductive activity. Rayleigh tests (*Z* statistics) determine whether there was significant seasonality.
(DOCX)

S3 Table Results of circular statistics for the occurrence of seasonality in fig initiation and fig production of *F*. *squamosa* in four study sites. Mean angle (or mean vector), or mean date, indicates average date of the fig initiation and fig production peak among individuals. Vector *r* indicates the extent of synchrony of reproductive activity. Rayleigh tests (*z*) determine whether there was statistically significant seasonality.
(DOCX)

S4 Table The full result shows correlations between phenology and meteorological factors for *Ficus squamosa*. (DOCX)
