# Supplementary figures and images for: Seasonality of Leaf and Fig Production in Ficus squamosa, a Fig Tree with Seeds Dispersed by Water
Source: PLoS One. 2016 Mar 24;11(3):e0152380. doi: 10.1371/journal.pone.0152380 (PMC4807038; doi:10.1371/journal.pone.0152380)

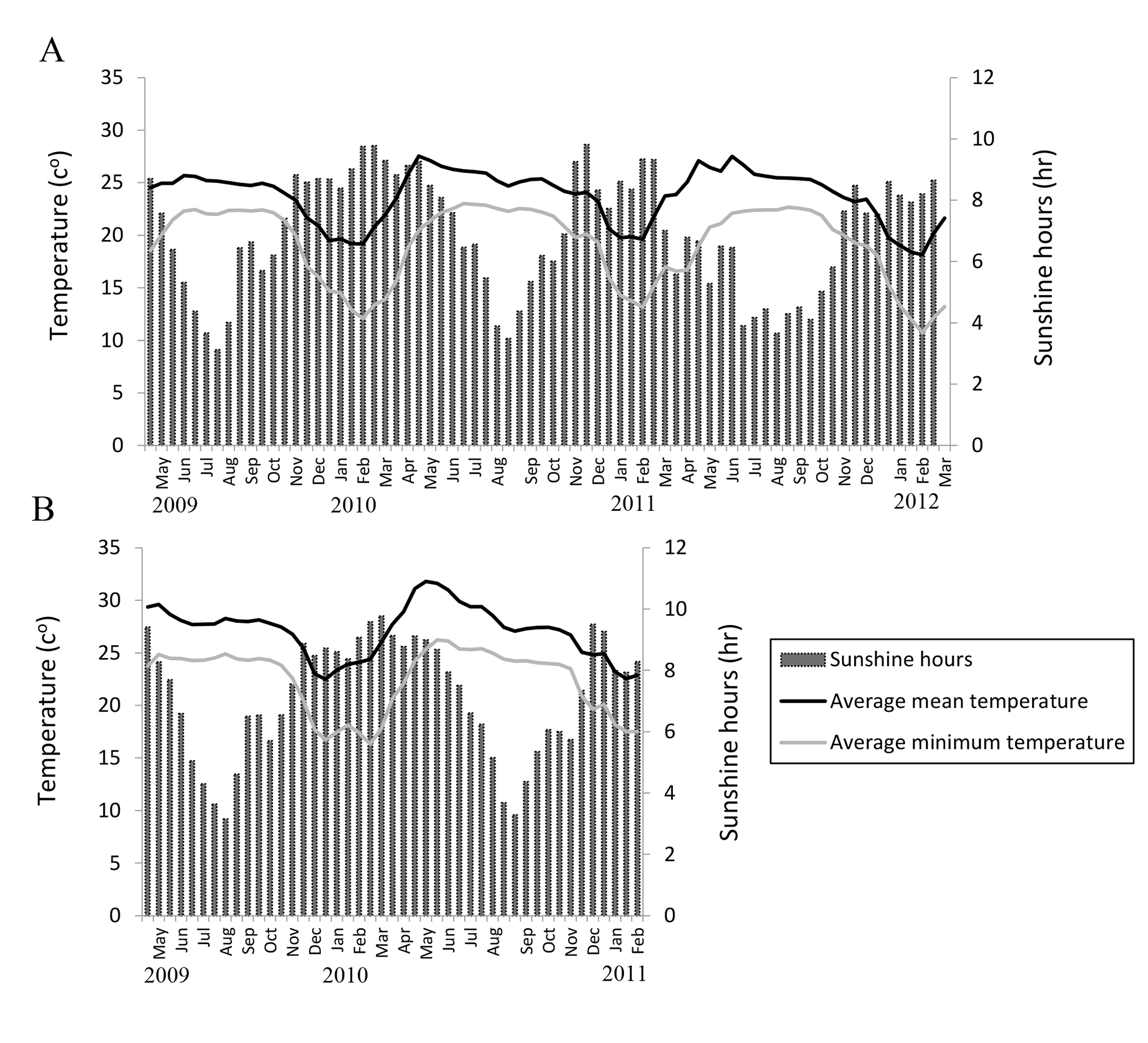

Supplement: S1 Fig — (A) the northern site; Mae Ka (MK) and Pang Dang Nai (PDN); (B) the sourthern sites Mae Sa (MS) and Mae Sa Noi (MSN). (Source: Thai Meteorology Department, 2012, Available: http://meteorology.hrdi.or.th/website, accessed 26 February 2013). (TIF) [file pone.0152380.s001.tif]

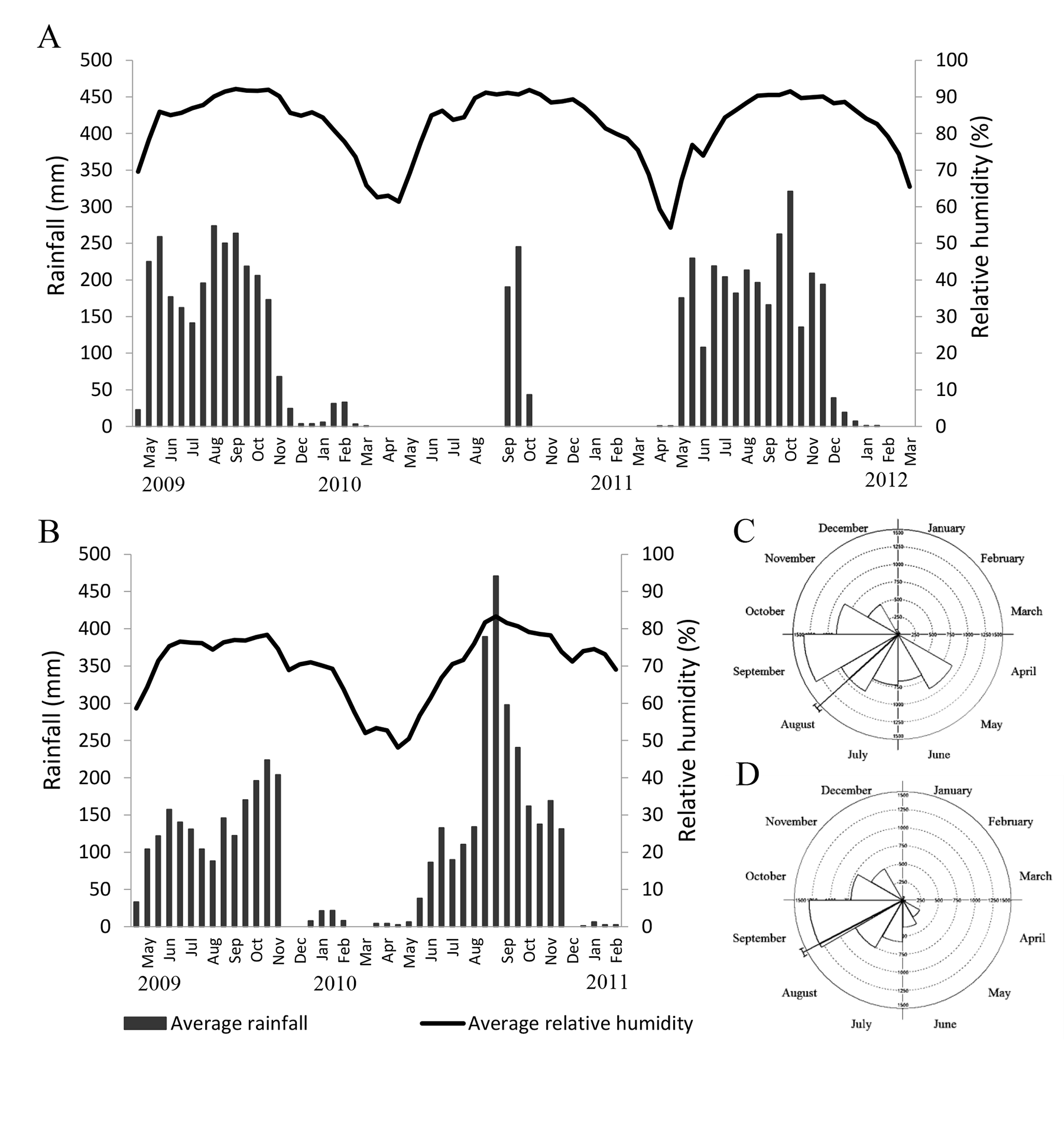

Supplement: S2 Fig — Monthly average rainfall and relative humidity; A, at Mae Ka (MK) and Pang Dang Nai (PDN); B, at Mae Sa (MS) and Mae Sa Noi (MSN); Circular histograms distributions showing the mean and distribution of rainfall in the study sites; C, MK and PDN sites; D, MS and MSN sites. (Source: Thai Meteorology Department, 2012, Available: http://meteorology.hrdi.or.th/website, accessed 26 February 2013). (TIF) [file pone.0152380.s002.tif]

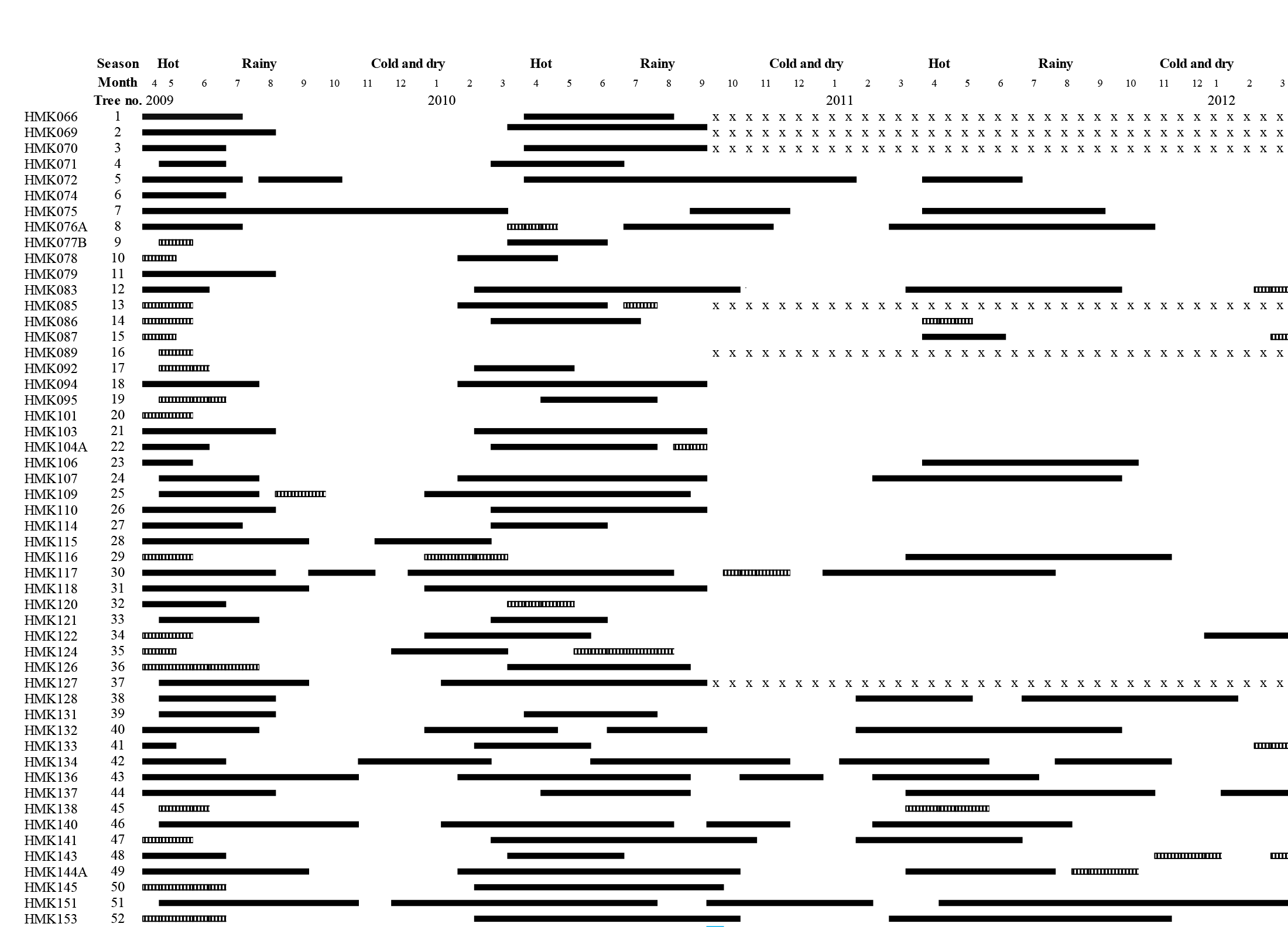

Supplement: S3 Fig — (Hatched bars is aborted crop and ‘x’ is the tree lost during the period of study) (TIF) [file pone.0152380.s003.tif]

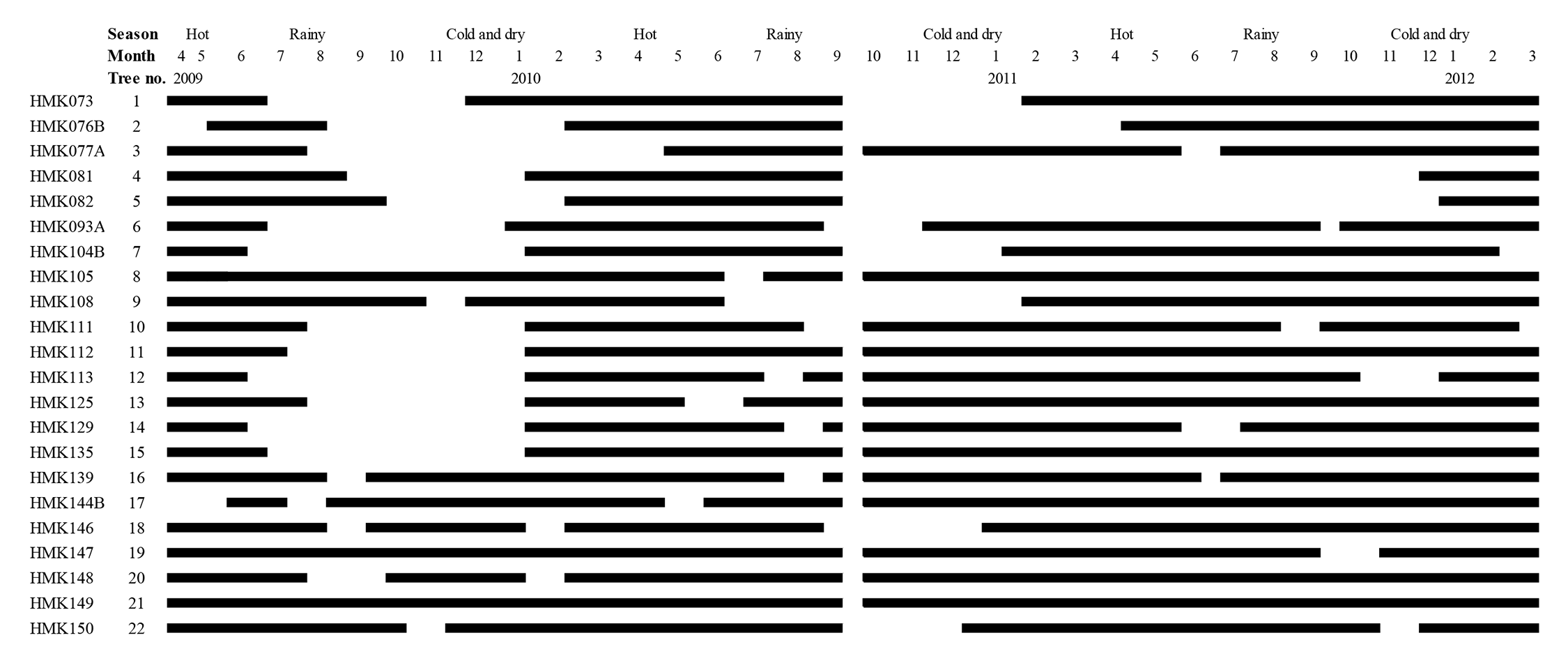

Supplement: S4 Fig — (TIF) [file pone.0152380.s004.tif]
