# Supplementary material for: Seasonality of Leaf and Fig Production in Ficus squamosa, a Fig Tree with Seeds Dispersed by Water
Source: PLoS One. 2016 Mar 24;11(3):e0152380. doi: 10.1371/journal.pone.0152380 (PMC4807038; doi:10.1371/journal.pone.0152380)
Supplement: S1 Table — (DOCX) [file pone.0152380.s005.docx]

**Table S1. Geological and environmental characteristics of the study sites.**

| **Study Site (stream)** | **Longitude** | **Latitude** | **Bed rock**  **(MacDonald *et al.* 2010)** | **Stream width (m)** |
| --- | --- | --- | --- | --- |
| **Huay Mae Ka**  **(HMK)** | 98º915´E | 19 º361´N | granite and  limestone | 3.5 - 12.0 |
| **Pang Dang Nai**  **(PDN)** | 99º039´E | 19 º348´N | granite  and limestone | 3.0 - 15.0 |
| **Mae Sa**  **(MS)** | 98º939´E | 18 º814´N | granite | 7.0 - 20.0 |
| **Mae Sa Noi**  **(MSN)** | 98º859´E | 18 º898´N | granite | 5.0 - 15.0 |
