# Supplementary material for: Seasonality of Leaf and Fig Production in Ficus squamosa, a Fig Tree with Seeds Dispersed by Water
Source: PLoS One. 2016 Mar 24;11(3):e0152380. doi: 10.1371/journal.pone.0152380 (PMC4807038; doi:10.1371/journal.pone.0152380)
Supplement: S2 Table — Mean angle (a), and mean date, indicates the months of the leafing peak. Vector r indicates the extent of synchrony of reproductive activity. Rayleigh tests (Z statistics) determine whether there was significant seasonality. (DOCX) [file pone.0152380.s006.docx]

**Table S2. Results of circular statistics analyses testing for the occurrence of seasonality in leaf initiation of *F. squamosa* at four sites.**

| Study site | Sex | Observation (*N*) | Mean  Angle (*a*) | Mean date | Length of mean vector (*r*) | Circular standard deviation | Rayleigh test (*Z*) | Raleigh test (*P*) |
| --- | --- | --- | --- | --- | --- | --- | --- | --- |
| MK | F | 1011 | 144.447^o^ | May | 0.189 | 104.53 ^o^ | 36.24 | <0.001 |
|  | M | 466 | 177.923 ^o^ | June | 0.068 | 132.82 ^o^ | 2.16 | 0.115 |
| PDN | F | 448 | 148.127 ^o^ | May | 0.177 | 106.70 ^o^ | 13.96 | <0.001 |
|  | M | 254 | 152.756 ^o^ | June | 0.223 | 99.29 ^o^ | 12.61 | <0.001 |
| MS | F | 367 | 199.838 ^o^ | July | 0.193 | 103.89 ^o^ | 13.70 | <0.001 |
|  | M | 118 | 162.149 ^o^ | June | 0.299 | 89.09 ^o^ | 10.52 | <0.001 |
| MSN | F | 270 | 173.519 ^o^ | June | 0.245 | 96.10 ^o^ | 16.20 | <0.001 |
|  | M | 179 | 266.785 ^o^ | September | 0.149 | 117.71 ^o^ | 3.99 | 0.018 |

Mean angle (*a*), and mean date, indicates the months of the leafing peak. Vector *r* indicates the extent of synchrony of reproductive activity. Rayleigh tests (*Z* statistics) determine whether there was significant seasonality.
