# Supplementary material for: Seasonality of Leaf and Fig Production in Ficus squamosa, a Fig Tree with Seeds Dispersed by Water
Source: PLoS One. 2016 Mar 24;11(3):e0152380. doi: 10.1371/journal.pone.0152380 (PMC4807038; doi:10.1371/journal.pone.0152380)
Supplement: S3 Table — Mean angle (or mean vector), or mean date, indicates average date of the fig initiation and fig production peak among individuals. Vector r indicates the extent of synchrony of reproductive activity. Rayleigh tests (z) determine whether there was statistically significant seasonality. (DOCX) [file pone.0152380.s007.docx]

**Table S3. Results of circular statistics for the occurrence of seasonality in fig initiation and fig production of *F. squamosa* in four study sites.**

|  | Study site | Sex | Observation (*N*) | Mean  Angle(*a*) | Mean date | Length of mean vector (*r*) | Circular standard deviation | Rayleigh test (*z*) | Raleigh test (*p*) |
| --- | --- | --- | --- | --- | --- | --- | --- | --- | --- |
| Fig  initiation | HMK | F | 296 | 129.568^o^ | May | 0.350 | 82.984 ^o^ | 36.33 | <0.001 |
|  |  | M | 208 | 96.524 ^o^ | April | 0.268 | 92.975 ^o^ | 14.94 | <0.001 |
|  | PDN | F | 157 | 136.645 ^o^ | May | 0.329 | 85.482 ^o^ | 16.95 | <0.001 |
|  |  | M | 148 | 115.295 ^o^ | April | 0.090 | 125.596 ^o^ | 1.21 | 0.298 |
|  | MS | F | 191 | 152.446 ^o^ | June | 0.563 | 61.458 ^o^ | 60.44 | <0.001 |
|  |  | M | 82 | 177.78 ^o^ | June | 0.099 | 123.334 ^o^ | 0.80 | 0.451 |
|  | MSN | F | 112 | 173.199 ^o^ | June | 0.646 | 53.590 ^o^ | 46.70 | <0.001 |
|  |  | M | 100 | 116.457 ^o^ | April | 0.243 | 96.503 ^o^ | 5.90 | 0.003 |
| Fig production | HMK | F | 12124 | 140.735 ^o^ | May | 0.430 | 74.418 ^o^ | 2243.82 | <0.001 |
|  |  | M | 7341 | 76.77 ^o^ | Mar | 0.291 | 90.047 ^o^ | 620.96 | <0.001 |
|  | PDN | F | 20505 | 141.467 ^o^ | May | 0.496 | 67.877 ^o^ | 5038.94 | <0.001 |
|  |  | M | 3912 | 115.727 ^o^ | April | 0.212 | 100.854 ^o^ | 176.51 | <0.001 |
|  | MS | F | 8032 | 166.933 ^o^ | June | 0.561 | 65.865 ^o^ | 2142.40 | <0.001 |
|  |  | M | 2652 | 121.487 ^o^ | May | 0.402 | 77.315 ^o^ | 429.32 | <0.001 |
|  | MSN | F | 8173 | 177.476 ^o^ | June | 0.729 | 45.589 ^o^ | 4339.36 | <0.001 |
|  |  | M | 2644 | 123.271 ^o^ | May | 0.391 | 78.543 ^o^ | 403.77 | <0.001 |

Mean angle (or mean vector), or mean date, indicates average date of the fig initiation and fig production peak among individuals. Vector *r* indicates the extent of synchrony of reproductive activity. Rayleigh tests (*z*) determine whether there was statistically significant seasonality.
