# Supplementary material for: Seasonality of Leaf and Fig Production in Ficus squamosa, a Fig Tree with Seeds Dispersed by Water
Source: PLoS One. 2016 Mar 24;11(3):e0152380. doi: 10.1371/journal.pone.0152380 (PMC4807038; doi:10.1371/journal.pone.0152380)
Supplement: S4 Table — (DOCX) [file pone.0152380.s008.docx]

**Table S4.** **The full result shows correlations between phenology and meteorological factors for *Ficus squamosa.***

| Average  temperature | Female | | | | Male | | | | Female | | | | Male | | | | |
| --- | --- | --- | --- | --- | --- | --- | --- | --- | --- | --- | --- | --- | --- | --- | --- | --- | --- |
|  | A-phase | | Total | | A-phase | | Total | | A-phase | | Total | | A-phase | | Total | | |
|  | *rho* | *p-value* | *rho* | *p-value* | *rho* | *p-value* | *rho* | *p-value* | *rho* | *p-value* | *rho* | *p-value* | *rho* | *p-value* | *rho* | | *p-value* |
|  | Huay Mae Ka (MK) | | | | | | | | Pang Dang Nai (PDN) | | | | | | | | |
| 10 preceding days | 0.323*^**^* | 0.006 | 0.488*^***^* | 0.000 | -0.175 | 0.142 | 0.004 | 0.972 | 0.172 | 0.148 | 0.338*^**^* | 0.004 | 0.166 | 0.165 | 0.263*^*^* | 0.026 | |
| 20 preceding days | 0.317*^**^* | 0.007 | 0.494*^***^* | 0.000 | -0.210 | 0.077 | -0.042 | 0.727 | 0.168 | 0.158 | 0.361*^**^* | 0.002 | 0.158 | 0.185 | 0.269*^*^* | 0.023 | |
| 30 preceding days | 0.258*^*^* | 0.029 | 0.453*^***^* | 0.000 | -0.258*^*^* | 0.029 | -0.111 | 0.353 | 0.133 | 0.267 | 0.340*^**^* | 0.003 | 0.140 | 0.241 | 0.225 | 0.057 | |
| 60 preceding days | 0.163 | 0.172 | 0.345*^**^* | 0.003 | -0.419*^***^* | 0.000 | -0.339 | 0.004 | 0.044 | 0.716 | 0.252*^*^* | 0.033 | 0.038 | 0.754 | 0.071 | 0.553 | |
| 10-day delay | 0.308*^**^* | 0.009 | 0.484*^***^* | 0.000 | -0.086 | 0.472 | 0.089 | 0.458 | 0.150 | 0.210 | 0.448*^***^* | 0.000 | 0.157 | 0.187 | 0.317*^**^* | 0.007 | |
|  | Mae Sa (MS) | | | | | | | | Mae Sa Noi (MSN) | | | | | | | | |
| 10 preceding days | 0.415*^**^* | 0.005 | 0.421*^**^* | 0.004 | 0.248 | 0.101 | 0.395*^**^* | 0.007 | 0.678*^***^* | 0.000 | 0.740*^***^* | 0.000 | 0.241 | 0.111 | 0.397*^**^* | | 0.007 |
| 20 preceding days | 0.404*^**^* | 0.006 | 0.411*^**^* | 0.005 | 0.192 | 0.206 | 0.320*^*^* | 0.032 | 0.661*^***^* | 0.000 | 0.744*^***^* | 0.000 | 0.183 | 0.228 | 0.343*^*^* | | 0.021 |
| 30 preceding days | 0.443*^**^* | 0.002 | 0.45*^**^* | 0.002 | 0.160 | 0.294 | 0.293 | 0.051 | 0.682*^***^* | 0.000 | 0.771*^***^* | 0.000 | 0.139 | 0.361 | 0.310*^*^* | | 0.038 |
| 60 preceding days | 0.457*^**^* | 0.002 | 0.465*^***^* | 0.001 | 0.034 | 0.826 | 0.141 | 0.356 | 0.678*^***^* | 0.000 | 0.794*^***^* | 0.000 | 0.008 | 0.959 | 0.167 | | 0.273 |
| 10-day delay | 0.297*^*^* | 0.048 | 0.330*^*^* | 0.027 | 0.261 | 0.084 | 0.381*^**^* | 0.010 | 0.508*^***^* | 0.000 | 0.559*^***^* | 0.000 | 0.245 | 0.105 | 0.387*^**^* | | 0.009 |

*rho: Spearman's rank correlation coefficient.*

**P* < 0.05*; **P* < 0.01*; ***P* < 0.001 (Spearman's rank correlation test).

Different temperature factor averaged over the 10, 20, 30, 60 preceding days, and with a 10-day delay were calculated.

| Minimun temperature | Female | | | | Male | | | | Female | | | | Male | | | | |
| --- | --- | --- | --- | --- | --- | --- | --- | --- | --- | --- | --- | --- | --- | --- | --- | --- | --- |
|  | A-phase | | Total | | A-phase | | Total | | A-phase | | Total | | A-phase | | Total | | |
|  | *rho* | *p-value* | *rho* | *p-value* | *rho* | *p-value* | *rho* | *p-value* | *rho* | *p-value* | *rho* | *p-value* | *rho* | *p-value* | *rho* | | *p-value* |
|  | Huay Mae Ka (MK) | | | | | | | | Pang Dang Nai (PDN) | | | | | | | | |
| 10 preceding days | 0.076 | 0.527 | 0.237*^*^* | 0.045 | -0.466*^***^* | 0.000 | -0.413*^***^* | 0.000 | 0.006 | 0.959 | 0.205 | 0.084 | -0.037 | 0.759 | -0.006 | 0.963 | |
| 20 preceding days | 0.018 | 0.878 | 0.173 | 0.145 | -0.509*^***^* | 0.000 | -0.505*^***^* | 0.000 | -0.026 | 0.831 | 0.121 | 0.311 | -0.078 | 0.514 | -0.082 | 0.496 | |
| 30 preceding days | -0.033 | 0.786 | 0.101 | 0.397 | -0.545*^***^* | 0.000 | -0.565*^***^* | 0.000 | -0.051 | 0.670 | 0.054 | 0.565 | -0.111 | 0.352 | -0.155 | 0.194 | |
| 60 preceding days | -0.116 | 0.331 | -0.046 | 0.699 | -0.624*^***^* | 0.000 | -0.684*^***^* | 0.000 | -0.063 | 0.597 | -0.084 | 0.483 | -0.163 | 0.172 | -0.297*^**^* | 0.001 | |
| 10-day delay | 0.075 | 0.532 | 0.277*^*^* | 0.019 | -0.410*^***^* | 0.000 | -0.359*^***^* | 0.000 | 0.070 | 0.561 | 0.278*^*^* | 0.018 | 0.033 | 0.782 | 0.056 | 0.643 | |
|  | Mae Sa (MS) | | | | | | | | Mae Sa Noi (MSN) | | | | | | | | |
| 10 preceding days | 0.428*^**^* | 0.003 | 0.409*^**^* | 0.005 | 0.089 | 0.562 | 0.176 | 0.248 | 0.639*^***^* | 0.000 | 0.747*^***^* | 0.000 | 0.084 | 0.583 | 0.197 | | 0.194 |
| 20 preceding days | 0468*^**^* | 0.001 | 0.489*^***^* | 0.001 | 0.055 | 0.720 | 0.146 | 0.340 | 0.673*^***^* | 0.000 | 0.782*^***^* | 0.000 | 0.047 | 0.761 | 0.163 | | 0.282 |
| 30 preceding days | 0.394*^**^* | 0.007 | 0.476*^***^* | 0.001 | -0.047 | 0.758 | 0.034 | 0.827 | 0.665*^***^* | 0.000 | 0.784*^***^* | 0.000 | -0.066 | 0.668 | 0.053 | | 0.728 |
| 60 preceding days | 0.223 | 0.141 | 0.320 | 0.032 | -0.233 | 0.123 | -0.222 | 0.142 | 0.438*^***^* | 0.001 | 0.625*^***^* | 0.000 | -0.251 | 0.097 | -0.197 | | 0.194 |
| 10-day delay | 0.401*^**^* | 0.006 | 0.430*^**^* | 0.003 | 0.125 | 0.412 | 0.223 | 0.141 | 0.629*^***^* | 0.000 | 0.706*^***^* | 0.000 | 0.114 | 0.455 | 0.223 | | 0.141 |

*rho : Spearman's rank correlation coefficient.*

**P* < 0.05*; **P* < 0.01*; ***P* < 0.001 (Spearman's rank correlation test).

Different temperature factor averaged over the 10, 20, 30, 60 preceding days, and with a 10-day delay were calculated.

| %Relative  humidity | Female | | | | Male | | | | Female | | | | Male | | | | |
| --- | --- | --- | --- | --- | --- | --- | --- | --- | --- | --- | --- | --- | --- | --- | --- | --- | --- |
|  | A-phase | | Total | | A-phase | | Total | | A-phase | | Total | | A-phase | | Total | | |
|  | *rho* | *p-value* | *rho* | *p-value* | *rho* | *p-value* | *rho* | *p-value* | *rho* | *p-value* | *rho* | *p-value* | *rho* | *p-value* | *rho* | | *p-value* |
|  | Huay Mae Ka (MK) | | | | | | | | Pang Dang Nai (PDN) | | | | | | | | |
| 10 preceding days | -0.246*^*^* | 0.037 | -0.333*^**^* | 0.004 | -0.457*^***^* | 0.000 | -0.650*^***^* | 0.000 | -0.210 | 0.077 | -0.341*^**^* | 0.003 | -0.311*^**^* | 0.008 | -0.519*^***^* | 0.000 | |
| 20 preceding days | -0.284*^*^* | 0.016 | -0.404*^***^* | 0.000 | -0.460*^***^* | 0.000 | -0.664*^***^* | 0.000 | -0.212 | 0.074 | -0.414*^***^* | 0.000 | -0.350*^**^* | 0.003 | -0.568*^***^* | 0.000 | |
| 30 preceding days | -0.315*^**^* | 0.007 | -0.454*^***^* | 0.000 | -0.445*^***^* | 0.000 | -0.672*^***^* | 0.000 | -0.193 | 0.105 | -0.442*^***^* | 0.000 | -0.354*^**^* | 0.002 | -0.592*^***^* | 0.000 | |
| 60 preceding days | -0.377*^**^* | 0.001 | -0.566*^***^* | 0.000 | -0.355*^**^* | 0.002 | -0.611*^***^* | 0.000 | -0.245*^*^* | 0.039 | -0.563*^***^* | 0.000 | -0.363*^**^* | 0.002 | -0.640*^***^* | 0.000 | |
| 10-day delay | -0.149 | 0.211 | -0.2121 | 0.074 | -0.492*^***^* | 0.000 | -0.660*^***^* | 0.000 | -0.093 | 0.438 | -0.234*^*^* | 0.045 | -0.218 | 0.066 | -0.411*^***^* | 0.000 | |
|  | Mae Sa (MS) | | | | | | | | Mae Sa Noi (MSN) | | | | | | | | |
| 10 preceding days | -0.276 | 0.067 | -0.318*^*^* | 0.033 | -0.281 | 0.062 | -0.415*^**^* | 0.005 | -0.096 | 0.533 | -0.002 | 0.988 | -0.294*^*^* | 0.050 | -0.401*^**^* | | 0.006 |
| 20 preceding days | -0.314*^*^* | 0.035 | -0.320*^*^* | 0.032 | -0.302*^*^* | 0.044 | -0.434*^**^* | 0.003 | -0.124 | 0.417 | -0.034 | 0.827 | -0.319*^*^* | 0.033 | -0.427*^**^* | | 0.003 |
| 30 preceding days | -0.314 | 0.022 | -0.371*^*^* | 0.012 | -0.307*^*^* | 0.041 | -0.450*^**^* | 0.002 | -0.163 | 0.284 | -0.058 | 0.706 | -0.310*^*^* | 0.038 | -0.444*^**^* | | 0.002 |
| 60 preceding days | -0.459*^**^* | 0.002 | -0.497*^***^* | 0.001 | -0.354*^*^* | 0.017 | -0.513*^***^* | 0.000 | -0.338*^*^* | 0.023 | -0.244 | 0.107 | -0.338*^*^* | 0.023 | -0.523*^***^* | | 0.000 |
| 10-day delay | -0.037 | 0.811 | 0.071 | 0.644 | -0.265 | 0.078 | -0.319*^*^* | 0.033 | 0.102 | 0.504 | 0.229 | 0.131 | -0.286 | 0.057 | -0.292 | | 0.052 |

*rho: Spearman's rank correlation coefficient.*

**P* < 0.05*; **P* < 0.01*; ***P* < 0.001 (Spearman's rank correlation test).

Different percent relative humidity factor averaged over the 10, 20, 30, 60 preceding days, and with a 10-day delay were calculated.

| Sunshine hours | Female | | | | Male | | | | Female | | | | Male | | | | |
| --- | --- | --- | --- | --- | --- | --- | --- | --- | --- | --- | --- | --- | --- | --- | --- | --- | --- |
|  | A-phase | | Total | | A-phase | | Total | | A-phase | | Total | | A-phase | | Total | | |
|  | *rho* | *p-value* | *rho* | *p-value* | *rho* | *p-value* | *rho* | *p-value* | *rho* | *p-value* | *rho* | *p-value* | *rho* | *p-value* | *rho* | | *p-value* |
|  | Huay Mae Ka (MK) | | | | | | | | Pang Dang Nai (PDN) | | | | | | | | |
| 10 preceding days | 0.068 | 0.568 | -0.028 | 0.815 | 0.478*^***^* | 0.000 | 0.471*^***^* | 0.000 | -0.161 | 0.177 | -0.148 | 0.217 | 0.109 | 0.364 | 0.173 | 0.145 | |
| 20 preceding days | 0.157 | 0.187 | 0.026 | 0.829 | 0.617*^***^* | 0.000 | 0.594*^***^* | 0.000 | -0.099 | 0.409 | -0.089 | 0.459 | 0.154 | 0.195 | 0.224 | 0.058 | |
| 30 preceding days | 0.194 | 0.102 | 0.089 | 0.456 | 0.636*^***^* | 0.000 | 0.613*^***^* | 0.000 | -0.042 | 0.725 | -0.073 | 0.545 | 0.193 | 0.105 | 0.271*^*^* | 0.021 | |
| 60 preceding days | 0.318*^**^* | 0.006 | 0.294*^*^* | 0.012 | 0.666*^***^* | 0.000 | 0.687*^***^* | 0.000 | 0.096 | 0.423 | 0.150 | 0.209 | 0.286*^*^* | 0.015 | 0.402*^***^* | 0.001 | |
| 10-day delay | 0.059 | 0.621 | -0.070 | 0.561 | 0.392*^***^* | 0.001 | 0.373*^***^* | 0.001 | 0.001 | 0.994 | -0.092 | 0.440 | 0.057 | 0.634 | 0.073 | 0.541 | |
|  | Mae Sa (MS) | | | | | | | | Mae Sa Noi (MSN) | | | | | | | | |
| 10 preceding days | 0.126 | 0.410 | 0.027 | 0.860 | 0.146 | 0.338 | 0.276 | 0.067 | -0.259 | 0.086 | -0.331*^*^* | 0.026 | 0.153 | 0.315 | 0.244 | | 0.107 |
| 20 preceding days | 0.133 | 0.384 | 0.003 | 0.987 | 0.060 | 0.697 | 0.161 | 0.579 | -0.299*^*^* | 0.046 | -0.392 | 0.008 | 0.070 | 0.649 | 0.153 | | 0.316 |
| 30 preceding days | 0.235 | 0.121 | 0.082 | 0.594 | 0.182 | 0.231 | 0.295*^*^* | 0.050 | -0.190 | 0.210 | -0.303*^*^* | 0.043 | 0.179 | 0.239 | 0.289 | | 0.054 |
| 60 preceding days | 0.286 | 0.057 | 0.184 | 0.227 | 0.314*^*^* | 0.036 | 0.420*^**^* | 0.004 | -0.034 | 0.824 | -0.156 | 0.305 | 0.310*^*^* | 0.038 | 0.416*^**^* | | 0.005 |
| 10-day delay | -0.131 | 0.391 | -0.289 | 0.055 | 0.072 | 0.640 | 0.085 | 0.289 | -0.392*^**^* | 0.008 | -0.528*^***^* | 0.000 | 0.098 | 0.524 | 0.076 | | 0.621 |

*rho : Spearman's rank correlation coefficient.*

NS *P* > 0.05*;*P* < 0.05*; **P* < 0.01*; ***P* < 0.001 (Spearman's rank correlation test).

Different sunshine hours factor averaged over the 10, 20, 30, 60 preceding days, and with a 10-day delay were calculated.

| Rainfall | Female | | | | Male | | | | Female | | | | Male | | | | |
| --- | --- | --- | --- | --- | --- | --- | --- | --- | --- | --- | --- | --- | --- | --- | --- | --- | --- |
|  | A-phase | | Total | | A-phase | | Total | | A-phase | | Total | | A-phase | | Total | | |
|  | *rho* | *p-value* | *rho* | *p-value* | *rho* | *p-value* | *rho* | *p-value* | *rho* | *p-value* | *rho* | *p-value* | *rho* | *p-value* | *rho* | | *p-value* |
|  | Huay Mae Ka (MK) | | | | | | | | Pang Dang Nai (PDN) | | | | | | | | |
| 10 preceding days | -0.002 | 0.986 | 0.043 | 0.723 | -0.376*^***^* | 0.001 | -0.262*^*^* | 0.026 | 0.095 | 0.429 | 0.248*^*^* | 0.036 | -0.202 | 0.089 | -0.144 | 0.227 | |
| 20 preceding days | -0.050 | 0.685 | -0.006 | 0.957 | -0.414*^***^* | 0.000 | -0.315*^**^* | 0.007 | 0.109 | 0.362 | 0.222 | 0.061 | -0.183 | 0.123 | -0.195 | 0.101 | |
| 30 preceding days | -0.141 | 0.238 | -0.065 | 0.568 | -0.437*^***^* | 0.000 | -0.356*^**^* | 0.002 | 0.092 | 0.444 | 0.160 | 0.180 | -0.212 | 0.074 | -0.266*^*^* | 0.024 | |
| 60 preceding days | -0.149 | 0.213 | -0.118 | 0.324 | -0.459*^***^* | 0.000 | -0.395*^***^* | 0.001 | 0.027 | 0.823 | 0.056 | 0.639 | -0.153 | 0.198 | -0.232*^*^* | 0.050 | |
| 10-day delay | 0.010 | 0.933 | 0.040 | 0.741 | -0.396*^***^* | 0.001 | -0.304*^**^* | 0.009 | 0.175 | 0.142 | 0.309*^**^* | 0.008 | -0.178 | 0.074 | -0.158 | 0.184 | |
| 30-day rolling totals | -0.141 | 0.238 | -0.065 | 0.586 | -0.437*^***^* | 0.000 | -0.357*^**^* | 0.002 | 0.092 | 0.444 | 0.160 | 0.180 | -0.212 | 0.135 | -0.266*^*^* | 0.024 | |
|  | Mae Sa (MS) | | | | | | | | Mae Sa Noi (MSN) | | | | | | | | |
| 10 preceding days | 0.025 | 0.873 | -0.042 | 0.784 | -0.048 | 0.757 | -0.107 | 0.485 | 0.316 | 0.035 | 0.423*^**^* | 0.004 | -0.068 | 0.658 | -0.084 | 0.584 | |
| 20 preceding days | 0.020 | 0.898 | -0.023 | 0.880 | -0.007 | 0.962 | -0.065 | 0.674 | 0.226 | 0.136 | 0.366*^*^* | 0.014 | -0.032 | 0.834 | -0.061 | 0.689 | |
| 30 preceding days | -0.053 | 0.728 | -0.134 | 0.380 | -0.093 | 0.542 | -0.172 | 0.258 | 0.097 | 0.528 | 0.243 | 0.107 | -0.108 | 0.482 | -0.158 | 0.301 | |
| 60 preceding days | -0.168 | 0.270 | -0.197 | 0.196 | -0.228 | 0.132 | -0.324*^*^* | 0.030 | -0.038 | 0.803 | 0.102 | 0.507 | -0.239 | 0.114 | -0.313*^*^* | 0.036 | |
| 10-day delay | 0.079 | 0.608 | 0.150 | 0.324 | 0.022 | 0.885 | 0.066 | 0.668 | 0.384*^**^* | 0.009 | 0.491*^***^* | 0.001 | -0.000 | 0.998 | 0.091 | 0.551 | |
| 30-day rolling totals | -0.070 | 0.649 | -0.152 | 0.318 | -0.095 | 0.535 | -0.178 | 0.243 | 0.088 | 0.564 | 0.238 | 0.116 | -0.107 | 0.486 | -0.162 | 0.287 | |

*rho : Spearman's rank correlation coefficient.*

**P* < 0.05*; **P* < 0.01*; ***P* < 0.001 (Spearman's rank correlation test).

Different rainfall factor averaged over the 10, 20, 30, 60 preceding days, a 10-day delay and with 30-day rolling totals were calculated.
